# Attention-deficit/hyperactivity disorder and chronic pain: a scoping review of epidemiology, clinical phenotypes, mechanisms, and treatment

**DOI:** 10.3389/fpsyt.2026.1837517

**Published:** 2026-07-13

**Authors:** Satoshi Kasahara, Shuntaro Aoki, Miwako Takahashi, Ryo Motoya, Takuma Sanpei, Taito Morita, Yusaku Fukada, Shin-Ichi Niwa

**Affiliations:** 1 Department of Anesthesiology and Pain Relief Center, The University of Tokyo Hospital, Tokyo, Japan; 2 Pain Management Center, Hoshi General Hospital, Fukushima, Japan; 3 Center for Medical Education and Career Development, Fukushima Medical University, Fukushima, Japan; 4 Department of Neuropsychiatry, Fukushima Medical University, Fukushima, Japan; 5 Institute for Quantum Medical Science, National Institutes for Quantum Science and Technology, Chiba, Japan; 6 School of Psychological Science, Health Sciences University of Hokkaido, Hokkaido, Japan; 7 Department of Psychiatry, Aizu Medical Center, Fukushima Medical University, Fukushima, Japan

**Keywords:** attention-deficit/hyperactivity disorder, central sensitization, chronic pain, comorbidity, dopamine, neurodevelopmental disorders, nociplastic pain, scoping review

## Abstract

**Introduction:**

Emerging evidence suggests a potential association between attention-deficit/hyperactivity disorder (ADHD) and chronic pain; however, extant findings are dispersed across disciplines and have not yet been comprehensively synthesized. This scoping review aimed to systematically map the epidemiological evidence, clinical phenotypes, proposed neurobiological mechanisms, and reported therapeutic interventions related to comorbid ADHD and chronic pain.

**Methods:**

A comprehensive literature search was conducted in PubMed, PsycINFO, and the Cochrane Library from database inception to December 28, 2025. Human studies examining the association between ADHD (diagnosed or symptom-defined) and chronic or recurrent pain were also included. Fifty studies met the eligibility criteria, including observational studies (cross-sectional and longitudinal), case reports and series, and one interventional study.

**Results:**

Epidemiological studies have consistently reported significant associations between ADHD symptoms or diagnoses and chronic pain in both the general population and clinical samples. Higher ADHD symptom burden was associated with greater pain severity and pain-related functional impairment. Comorbidity was observed not only in widespread pain syndromes, such as fibromyalgia, but also in site-specific conditions, including chronic low back pain, orofacial pain, and migraine. Proposed mechanisms involve dopaminergic and noradrenergic dysfunction affecting motor regulation, sensory processing, and descending pain modulation systems. Several case-based reports have described improvements in pain outcomes after ADHD-targeted pharmacotherapy. However, controlled trials remain scarce.

**Discussion:**

Current evidence suggests that ADHD traits may be relevant in a subset of individuals with chronic pain, particularly those with treatment-resistant presentations. Although causality and treatment efficacy remain unconfirmed, consideration of neurodevelopmental characteristics may enhance clinical assessments and inform future research on individualized mechanism-based treatment strategies.

## 1 Introduction

Chronic pain is a major public health concern affecting hundreds of millions of individuals worldwide; it is associated with substantial reductions in quality of life (QOL) and significant socioeconomic burden (1–3). In 2017, the International Association for the Study of Pain (IASP) introduced the concept of nociplastic pain, defined as pain arising from altered nociception in the absence of clear evidence of tissue damage or disease of the somatosensory system (4). Although this framework has advanced the understanding of centrally mediated pain mechanisms, a considerable proportion of patients continue to experience persistent symptoms despite standard treatments, including conventional analgesics and antidepressants (5–7). This treatment resistance suggests that additional comorbidities or neurobiological factors may contribute to symptom persistence and functional impairment (8, 9). The present review is not restricted to a specific mechanistic pain subtype; rather, it addresses chronic pain broadly while acknowledging that nociplastic mechanisms may be relevant in some cases.

Attention-deficit/hyperactivity disorder (ADHD) has recently emerged as a potential comorbid condition of interest in populations with chronic pain. ADHD is a neurodevelopmental disorder characterized by inattention, hyperactivity, and impulsivity (10); it is estimated to persist into adulthood in approximately 2.5%–3.4% of the general population (11, 12). Accumulating evidence suggests that ADHD symptoms or diagnoses may be more prevalent among individuals with chronic pain conditions, including fibromyalgia, chronic low back pain, migraines, and orofacial pain, than among the general population (13–17).

Several hypotheses have been proposed to explain this association. The dysregulation of dopaminergic and noradrenergic neurotransmitter systems has been implicated in both ADHD and pain modulation (18–20). These systems are involved not only in attentional control but also in descending pain inhibitory pathways and sensory processing (18, 21, 22). Neuroimaging studies have reported altered activity in the default mode network (DMN)-related regions in patients with pain who also exhibit ADHD symptoms, suggesting potential interactions between attentional networks and pain processing systems (23). Although these findings are preliminary, they point to the possibility of shared neurobiological substrates.

Adult ADHD is frequently underdiagnosed from a clinical perspective (24), and a similar under-recognition may occur in chronic pain settings (25). Case reports and series have described improvements in pain symptoms following pharmacological treatment targeting ADHD in individuals whose pain had been refractory to conventional analgesic strategies (26–28). Although such evidence is limited and primarily descriptive, it highlights the need for systematic evaluation of this comorbidity.

The literature examining the relationship between ADHD and chronic pain is distributed across multiple disciplines, including psychiatry, rheumatology, dentistry, and pain medicine. However, despite growing interest, it has not yet been comprehensively synthesized. In some cases, existing reviews have focused only on pediatric populations, leaving our understanding of the full lifespan perspective rather limited (29).

Therefore, the aim of this scoping review was to systematically map the existing evidence on the association between ADHD (diagnosed or symptom-defined) and chronic or recurrent pain across the lifespan. Specifically, we sought to characterize the epidemiological patterns, clinical phenotypes, proposed pathophysiological mechanisms, and reported treatment approaches. In addition, we aimed to identify knowledge gaps and methodological limitations in the current literature to inform future research priorities. To our best knowledge, this is the first scoping review to integrate epidemiological evidence, clinical phenotypes, proposed mechanisms, and treatment-related findings on the associations between ADHD and chronic or recurrent pain across both pediatric and adult populations.

## 2 Methods

### 2.1 Study design and protocol

This scoping review was conducted in accordance with the methodological framework originally proposed by Arksey and O’Malley (30) and further refined by Levac et al. (31). Reporting followed the Preferred Reporting Items for Systematic reviews and Meta-Analyses extension for Scoping Reviews (PRISMA-ScR) guidelines (32). The review was not prospectively registered, consistent with the exploratory nature of scoping reviews, which aim to comprehensively map the literature rather than synthesizing effect estimates.

### 2.2 Search strategy

We performed a comprehensive search in PubMed, PsycINFO, and the Cochrane Library to identify literature from the time of database inception to December 28, 2025.

The search strategy involved combining controlled vocabulary and free-text keywords related to ADHD and pain. The ADHD-related terms included “attention-deficit/hyperactivity disorder,” “ADHD,” and names of commonly used assessment scales (e.g., ASRS, CAARS, and SNAP-IV), while the pain-related terms included “chronic pain,” “fibromyalgia,” “headache,” and other pain conditions.

The full PubMed search strategy is provided in the **Supplementary Material**. The search strategies for other databases were adapted according to the PubMed search strategy.

In addition to the database search, a manual re-screening of reference lists and relevant journal sources was also conducted to ensure comprehensive coverage of the literature. During this process, we identified three additional eligible studies published in journals that are not indexed in the searched databases.

### 2.3 Eligibility criteria

Studies were included if they met the following criteria:

#### 2.3.1 Participants

Human participants of any age (children, adolescents, or adults) who were either:

- Clinically diagnosed with ADHD, or
- Assessed as having ADHD symptoms

We also considered studies involving individuals with chronic pain or general population samples in which both ADHD and chronic pain variables were examined.

#### 2.3.2 Concept

Studies were required to focus on the relationship between ADHD and chronic pain. Specifically, the eligible studies examined at least one of the following:

- Prevalence of comorbidity
- Associations or correlations between ADHD and chronic pain
- Shared neurobiological or psychosocial mechanisms (e.g., dopaminergic or noradrenergic system dysfunction, descending pain inhibitory pathways, sensory gating)
- Treatment effects, including the impact of ADHD treatment on pain outcomes or impact of pain treatment on ADHD symptoms

ADHD was defined based on Diagnostic and Statistical Manual of Mental Disorders (DSM) (10) or International Classification of Diseases (ICD) criteria (33) or assessed using validated rating scales (e.g., Adult ADHD Self-Report Scale (ASRS), Wender Utah Rating Scale (WURS)).

Chronic pain was defined as pain persisting for ≥3 months or explicitly described as recurrent or persistent. Pain assessment could be based on physician diagnosis or self-report. For headache conditions, only studies that explicitly described chronic or recurrent headaches were included.

#### 2.3.3 Context

Both clinical settings (e.g., pain clinics, psychiatric services) and population-based studies were eligible.

#### 2.3.4 Types of evidence

Primary human studies were included, comprising:

- Observational studies (cross-sectional, longitudinal, or cohort designs)
- Interventional studies (randomized controlled trials (RCTs) or non-randomized single-arm studies)
- Case reports and series

The following were excluded:

- Review articles
- Animal studies
- Conference abstracts

#### 2.3.5 Language

Only articles published in English were included.

### 2.4 Study selection

A total of 11,150 records identified through database searches and three additional records found through manual searches were imported into Rayyan (34), a web-based platform designed to support systematic and scoping reviews.

After excluding 269 duplicate records, 10,884 studies remained for screening. Two researchers independently conducted the selection process. Any disagreements between the researchers were resolved through discussion and consensus.

**Figure 1:**
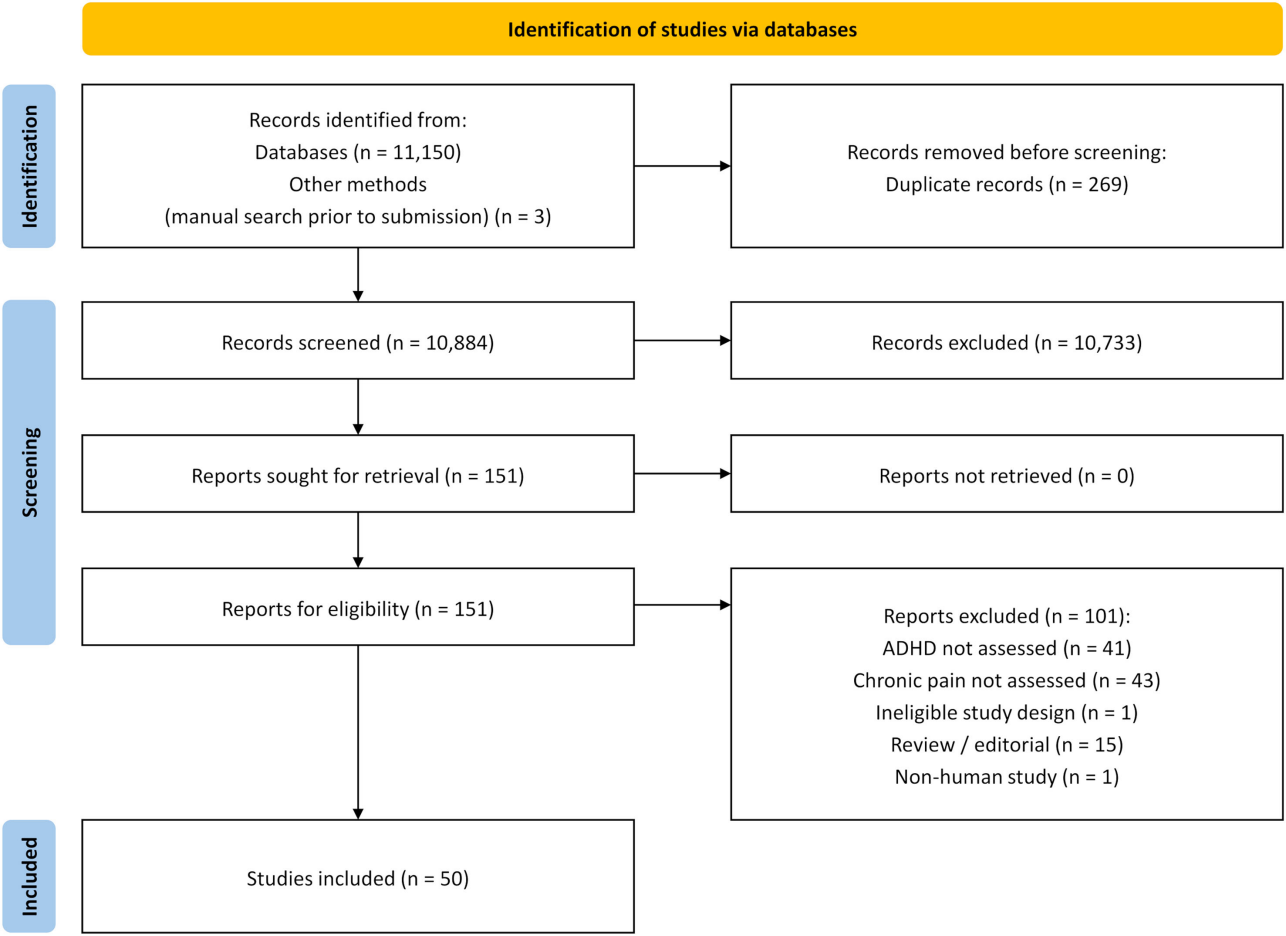
PRISMA flow diagram of study selection. In total, 11,150 records were identified through database searching. After removing duplicates (n = 269), 10,881 records remained. Additionally, three records identified through manual searches prior to manuscript submission were included, resulting in 10,884 records screened at the title and abstract levels. Of these, 151 were assessed for full-text eligibility. After full-text review, 101 reports were excluded for the following reasons: absence of ADHD assessment (n = 41), absence of chronic pain assessment (n = 43), review articles or editorials (n = 15), ineligible study design (n = 1), or non-human research (n = 1). Ultimately, 50 studies were included in this scoping review. ADHD, attention-deficit/hyperactivity disorder.

The reasons for exclusion were categorized and recorded and are presented in the PRISMA flow diagram (**Figure 1**).

#### 2.4.1 Level 1: title and abstract screening

To ensure consistent application of the eligibility criteria, pilot screening was conducted using 200 randomly selected records in Rayyan’s blinded mode. Discrepancies occurred in five cases (2.5%). After unblinding, the reviewers discussed these cases and clarified their interpretations of the inclusion criteria.

The remaining records were then divided between the two reviewers and screened independently.

#### 2.4.2 Level 2: full-text screening

In total, 151 articles advanced to full-text review. A second pilot screening was conducted in blinded mode using 20% of these articles (n = 29). Discrepancies were identified in nine cases (31%), prompting further clarification and refinement of the eligibility criteria.

Subsequently, both reviewers independently assessed all full-text articles. Disagreements were resolved through discussions.

A total of 50 studies were ultimately included in the review.

### 2.5 Data charting and synthesis

Data extraction was performed by the first reviewer (SK) using a standardized data charting form developed *a priori*, and independently verified for accuracy by the second reviewer (SA). Disagreements were resolved through discussions.

The data charting form was pilot-tested on a subset of studies and refined as necessary.

The following information was extracted from the 50 included studies:

- Author(s)
- Year of publication
- Country
- Study design
- Participant characteristics (sample size, age, sex)
- Type of pain (diagnostic category)
- ADHD assessment method (diagnostic criteria or rating scale)
- Key findings (e.g., prevalence rates, strength of associations, treatment outcomes)

Extracted data were categorized into four thematic domains and synthesized narratively:

- Epidemiology and prevalence
- Clinical phenotypes
- Pathophysiological mechanisms
- Therapeutic interventions and outcomes

As this scoping review aimed to map the breadth and characteristics of the evidence, a formal risk-of-bias assessment was not performed. To contextualize the strengths and limitations of the included studies, evidence levels and key study limitations are presented in **Table 1**.

**Table 1:** Mechanistic hypotheses and therapeutic interventions in ADHD-related chronic pain (n = 22).

Author (year)	Study design	Population/pain condition	ADHD assessment	Proposed mechanism/pathophysiology	Intervention	Outcome	Level of evidence	Key limitations
Young & Redmond (2007) (13)	Case report	N = 1; Adult female with ADHD (47 years old) Pain: Fibromyalgia and chronic fatigue	Clinical interview and standardized symptom checklists (DSM-IV criteria)	Sensory–attentional system – sensory gating deficit	Psychostimulants (Adderall XR)	Pain ↓; fatigue ↓; cognitive dysfunction ↓	Very low	Single case; no control; unclear ADHD diagnosis
Vorobeychik & Acquadro (2008) (73)	Case report	N = 1; Adult female with ADHD (65 years old) Pain: Fibromyalgia	Previously established clinical diagnosis; AADDC	Descending pain modulation system – noradrenergic inhibitory pathway	ATX (80 mg/day)	Pain intensity ↓ (~60%); function ↑; sleep ↑; mood ↑	Very low	Single case; no control
Stray et al. (2013) (53)	Cross-sectional	N = 48 (25 cases/23 controls); Adults with or without ADHD (mean age = 32 years for cases) Pain: Widespread pain	Clinical diagnosis; M.I.N.I. (controls)	Motor system – increased muscle tone and impaired motor inhibition	–	Pain ↑; muscle tone ↑ in the ADHD group	Low	No causal inference
Wiwe Lipsker et al. (2018) (50)	Case report	N = 1; Female child with ASD and ADHD (6 years old) Pain: Idiopathic chronic pain	Clinical diagnosis, DSM-IV-TR (pediatric clinic)	Sensory over-responsivity and alterations in cognitive pathways for pain processing	Parent training (ACT-based) and MP	Pain intensity/frequency ↓; function ↑; parental distress ↓	Very low	Single case; no control; combination therapy
Balter et al. (2021) (65)	Interventional (single-arm trial)	N = 47 (70.2% female); Children and adolescents with chronic pain (8–18 years old, mean age = 14.8 years) Pain: Chronic pain	Conners-3 (parent-report)	Sensory–attentional system – neurodevelopmental traits influencing behavioral regulation	ACT (17 sessions)	Pain interference ↓; treatment outcome was NOT associated with ADHD symptoms (but moderated by autistic traits)	Low	No control group; small sample
Kasahara et al. (2021) (49)	Historical analysis (Letter to the Editor)	N = 1 (Historical figure); Adult female with ADHD (Margaret Mitchell) Pain: Fibromyalgia	Retrospective biographical review, DSM-5 (hypothetical)	Central nervous system dysfunction – Conceptual interpretation of ADHD traits in fibromyalgia	–	Hypothesis-generating reinterpretation of ADHD-related pain and cognitive impairment (“fibrofog”)	Very low	Hypothesis-generating; no empirical data
Wiwe Lipsker et al. (2021) (59)	Cross-sectional	N = 146 (69.9% female); Children and adolescents with chronic pain (8–17 years old, mean age = 14.6 years) Pain: Chronic pain	Conners-3 (parent-report) and SRS for autistic traits	Insomnia and psychological inflexibility	–	Pain interference ↑; depression ↑; QoL ↓ (mediated by insomnia and psychological inflexibility)	Low	No causal inference; self-reported ADHD
Kasahara et al. (2022) (37)	Historical analysis (Case report)	N = 1 (Historical figure); Adult male with ADHD (John F. Kennedy) Pain: Chronic low back pain (centralized pain)	Retrospective biographical review, DSM-5 (hypothetical)	Central nervous system dysfunction (dopaminergic/noradrenergic) – Conceptual interpretation of centralized pain with ADHD traits	–	Hypothesis-generating reinterpretation of centralized pain related to ADHD traits	Very low	Hypothesis-generating; no empirical data
Kasahara et al. (2022) (60)	Case report	N = 1; Adult male with ASD and ADHD (46 years old) Pain: Atypical odontalgia	CAARS-S/O, DSM-5	Sensory hyper-reactivity (ASD irritability) and impaired prefrontal filtering	RIS (1 mg/day) + ATX (120 mg/day)	Pain intensity (NRS) ↓ (from 7 to 4); anger/irritability ↓; PCS ↓; returned to work	Very low	Single case; no control; combination therapy
Bozkurt & Balta (2023) (72)	Cross-sectional	N = 123 (26.8% female, 82 cases/41 controls); Children (8–13 years old, median age = 10 years) Pain: Pain threshold study	DSM-5; K-SADS-PL; CPRS-R:S	Descending pain modulation system – catecholaminergic dysregulation affecting pain thresholds	MP - observational comparison	Pain threshold ↓ in ADHD; MP treatment normalized pain thresholds	Low	No causal inference; surrogate outcome
Kasahara et al. (2023) (64)	Case report	N = 1; Adolescent female with ASD and ADHD (15 years old) Pain: ADPKD-related chronic back pain	Conners-3 (self/parent), DSM-5	CFTR–dopamine receptor interaction; frontal hypoperfusion and insular hyperperfusion	GF (4 mg/day) + MP (18 mg/day)	Pain ↓; frontal CBF ↑ (insular CBF ↓); blood pressure controlled; returned to school	Very low	Single case; no control; combination therapy
Kasahara et al. (2023) (61)	Case report	N = 1; Older adult male with ADHD (80 years old) Pain: oral dysesthesia and chronic low back pain	CAARS-S/O, DSM-5	Prefrontal and limbic neurotransmission (dopaminergic dysfunction in reward circuits); frontal hypoperfusion	ATX (40–120 mg/day) + Pramipexole (0.25 mg/day)	Pain ↓ (remission of 25-year chronic pain); prefrontal CBF ↑; returned to work; improved family relations	Very low	Single case; no control; combination therapy
Kasahara et al. (2023) (16)	Retrospective cohort study (Case series)	N = 25 (80.0% female); Adults with ADHD (mean age = 57.6 years) Pain: Intractable idiopathic orofacial pain (BMS/PIDAP/PIFP)	CAARS-S/O, DIVA-2.0 (DSM-5 criteria)	Central sensitization – dopaminergic/prefrontal dysfunction affecting descending pain modulation and reward systems	Algorithm-based pharmacotherapy (MP, ATX, APZ, and Clonidine)	High ADHD prevalence (83.3%); Pain intensity (NRS) ↓; PCS ↓; HADS-A/D ↓ (achieved MCID)	Very low	No control; combination therapy
Kasahara et al. (2023) (28)	Case report	N = 1; Older adult female with ADHD (65 years old) Pain: persistent idiopathic facial pain (PIFP)	M.I.N.I., CAARS-S/O, DSM-5	Descending pain modulation system – dopaminergic hypofunction	MP (54 mg/day) + APZ (6 mg/day)	Pain behavior ↓ (remission); pain intensity (NRS) ↓; EQ-5D ↑; PCS ↓; returned to work	Very low	Single case; no control; combination therapy
Kasahara et al. (2023) (27)	Case report	N = 1; Adult female with PTSD and ADHD (56 years old) Pain: Chronic pain (facial, headache, right upper extremity)	CAARS-S/O, DSM-5	Prefrontal cortex dysfunction (NA/DA imbalance); frontal and striatal hypoperfusion	ATX (40–120 mg/day)	Pain intensity (NRS) ↓; PTSD symptoms ↓; HADS-A/D ↓; PCS ↓; prefrontal/striatal CBF ↑; returned to work	Very low	Single case; no control; long treatment course (cannot rule out natural recovery of PTSD)
Zain et al. (2023) (26)	Case report	N = 1; Adult male with ADHD (43 years old) Pain: chronic idiopathic pain (chest, back, headache)	ADHD-RS-IV, DSM-IV	Descending pain modulation system – dopaminergic dysfunction and increased pain sensitivity	MP (18–72 mg/day)	Pain ↓ (NRS 0/10; remission of 15-year chronic pain); inattention ↓; functional improvement (stable for 7 years)	Very low	Single case; no control
Kasahara et al. (2024) (62)	Case report	N = 1; Adult female with major depression, ADHD, and ASD (51 years old) Pain: Abdominal nociplastic pain	CAARS-S/O, DSM-5	Central sensitization; anterior cingulate/prefrontal cortex dysfunction; psychosocial factors (dysfunctional family dynamics)	MP (54 mg/day) + Venlafaxine (225 mg/day)	Abdominal pain ↓; depression/anxiety ↓; central sensitization (CSI-9) ↓; anterior cingulate/prefrontal CBF ↑; improved family dynamics (MPI: DYS to ID); returned to work	Very low	Single case; no control; combination therapy; long treatment course (cannot rule out natural recovery)
Takahashi, Kasahara et al. (2024) (23)	Retrospective cohort study	N = 65 (53.8% female); Adults with nociplastic pain and ADHD (mean age = 53.0 years) Pain: Nociplastic pain	CAARS-S, DSM-5	Brain network alteration (DMN hyperactivity) – precuneus, insular, and thalamic hyperperfusion	Algorithm-based pharmacotherapy (MP, ATX, APZ, Clonidine)	Pain intensity (NRS) ↓; CGI-S ↓; HADS-A/D ↓; PCS ↓; precuneus CBF normalization	Very low	Retrospective; no control; combination therapy
Udal et al. (2024) (54)	Cross-sectional	N = 121 (65.3% female); Adult psychiatric outpatients with or without chronic pain (18–66 years old, mean age 32.4 years) Pain: Chronic pain	M.I.N.I.-Plus, DIVA 2.0	Muscular dysregulation (high muscle tone and motor inhibition problems)	–	ADHD predicted axial pain; pain intensity correlated with muscular dysregulation (high muscle tone)	Low	No causal inference
Endres et al. (2025) (41)	Case report	N = 1; Adult female with ADHD and tics (36 years old) Pain: Severe pain (fibromyalgia and somatoform pain disorder)	CAARS, DSM-IV	Neuroimmune mechanism – anti-thalamus antibodies and CSF dopamine abnormalities	High-dose steroids (immunotherapy)	Pain ↓ minimal after immunotherapy (limited response)	Very low	Single case; no control
Martínez-González (2025) (36)	Cross-sectional	N = 151 (57.6% female, 75 cases/76 controls); Adolescents and young adults with or without neurodevelopmental disorders (ASD, ADHD, LD) (mean age 21.9 years for cases) Pain: Unspecified pain and gastrointestinal symptoms	Self-/caregiver- Previously established diagnosis (via questionnaire)	Sensory processing sensitivity (sensory hyper-reactivity)	–	Pain severity ↑ in ADHD (compared to controls); sensory hyper-reactivity strongly associated with pain and gastro-intestinal symptoms	Low	No causal inference
Takahashi, Kasahara et al. (2025) (63)	Retrospective cohort study (Case series)	N = 14 (85.7% female); Adults with treatment-resistant BMS (mean age 60.0 years) Pain: Burning mouth syndrome	CAARS-S/O, DIVA-2.0 (DSM-5 criteria)	Central sensitization – dopaminergic/noradrenergic dysfunction; frontal hypoperfusion and ACC/insular/precuneal hyperperfusion	Algorithm-based pharmacotherapy (MP, ATX, GF, APZ, Venlafaxine/Duloxetine)	High ADHD prevalence (92.9%); Pain intensity (NRS) ↓; HADS-A/D ↓; PCS ↓; brain perfusion abnormalities improved	Very low	Retrospective; no control; combination therapy

This table summarizes clinical and observational studies exploring potential pathophysiological mechanisms and treatment approaches in individuals with ADHD and chronic pain. The proposed mechanisms are organized according to a conceptual framework involving sensory–attentional processing, descending pain modulation, motor regulation, and brain network alterations. Pharmacological and non-pharmacological interventions reported in the literature are also summarized together with their clinical outcomes.

AADDC, Adult Attention Deficit Disorder Criteria; ACT, acceptance and commitment therapy; ACC, anterior cingulate cortex; ADHD, attention-deficit/hyperactivity disorder; ADHD-RS, ADHD Rating Scale; ADPKD, autosomal dominant polycystic kidney disease; APZ, aripiprazole; ASD, autism spectrum disorder; ATX, atomoxetine; BMS, Burning mouth syndrome; CAARS-S/O, Conners’ Adult ADHD Rating Scales Self-report/Observer-rated; CBF, cerebral blood flow; CFTR, cystic fibrosis transmembrane conductance regulator; CGI-S, clinical global impression severity; CPRS-R:S, Conners’ Parent Rating Scale–Revised Short form; CSF, cerebrospinal fluid; CSI-9, central sensitization inventory-9; DA/NA, dopamine/noradrenaline; DIVA, Diagnostic Interview for ADHD in Adults; DSM-IV(-TR)/5, Diagnostic and Statistical Manual of Mental Disorders, Fourth Edition (Text Revision)/Fifth Edition; DMN, default mode network; DYS, dysfunctional; EQ-5D, EuroQol 5-Dimension questionnaire; GF, guanfacine; HADS-A/D, Hospital Anxiety and Depression Scale, subscale for assessing anxiety/depression; ID, interpersonally distressed; K-SADS-PL, Kiddle Schedule for Affective Disorders and Schizophrenia for School-Age Children—Present and Lifetime Version; LD, Learning Difficulties; MCID, Minimum clinically important difference; M.I.N.I., Mini-International Neuropsychiatric Interview; MP, methylphenidate; MPI, Multidimensional Pain Inventory; NRS, numerical rating scale; PCS, Pain Catastrophizing Scale; PIDAP, Persistent idiopathic dentoalveolar pain; PIFP, Persistent idiopathic facial pain; PTSD, post-traumatic stress disorder; QoL, quality of life; RIS, risperidone; SRS, Social Responsiveness Scale. Evidence levels were assigned qualitatively based on the study design and methodological characteristics.

## 3 Results

The study selection process is illustrated in **Figure 1**. An overview of the included studies is presented in **Tables 1** and **2** (13, 14, 16, 17, 20, 23, 25–28, 35–74).

**Table 2:** Epidemiological studies examining the prevalence and association between ADHD and chronic pain (n = 28).

Author (year)/country	Study population (N)	Pain condition	ADHD assessment	Key findings (prevalence and association)
Holmberg & Hjern (2006) Sweden (45)	N = 516 (48.8% female); Fourth-grade children (10 years old)	Recurrent abdominal pain (RAP), headache (self-reported)	Structured interview (DSM-IV), Parent and teacher Conners Scale	ADHD was associated with recurrent abdominal pain (adjusted RR = 2.2) but not with headache.
Reyero et al. (2011) Spain (40)	N = 399 (201 cases/198 controls); Adult women	Fibromyalgia (ACR criteria)	Semi-structured interview (DSM-IV), WURS	A higher prevalence of a childhood history of ADHD was observed in fibromyalgia patients than in controls (32.3% vs. 2.5%; OR = 18.45).
Mangerud et al. (2013) Norway (68)	N = 566 (54.2% female, 216 with ADHD); Adolescent psychiatric patients (13–18 years old, mean age = 15.7 years)	Chronic pain and multi-site pain (self-reported)	Clinical diagnosis (ICD-10)	Chronic pain was reported by 65.9% of patients with ADHD. Comorbid mood/anxiety disorders further increased the risk of pain-related disability.
Derksen et al. (2015) The Netherlands (67)	N = 156; Adults with fibromyalgia	Fibromyalgia (ACR 1990 criteria)	Clinical diagnostic interview	Adult ADHD was diagnosed in 25% of the fibromyalgia patients, which is significantly higher than the general population prevalence (2.1%).
Fuller-Thomson et al. (2016) Canada (51)	N = 3908; Adult women (20–39 years old)	Chronic pain (self-reported)	Self-reported previous diagnosis	Women with ADHD had significantly higher odds of experiencing chronic pain compared to controls (OR = 3.86). They also showed higher odds of comorbid mental health problems (e.g., depression, anxiety, and suicidal ideation).
Stickley et al. (2016) England (43)	N = 7403 (51.4% female); General population adults (≥16 years old, mean age = 46.3 years)	Extreme pain interference (self-reported)	ASRS Screener	ADHD symptoms were associated with significantly higher odds of extreme pain (OR = 3.15). This association was attenuated but remained significant after adjusting for common mental disorders (adjusted OR = 1.64).
Kutuk et al. (2018) Turkey (39)	N = 228 (117 cases/111 controls); Children and adolescents (6–18 years old, median age = 11 years for cases)	Migraine and tension-type headache (ICHD-3 beta criteria), recurrent abdominal pain (clinical diagnosis)	DSM-5, K-SADS-PL	Migraine was nearly three times more frequent in children with ADHD compared to controls (26.5% vs. 9.9%; OR = 3.3), and recurrent abdominal pain was also significantly higher. Furthermore, migraine and recurrent abdominal pain were also significantly more prevalent among the mothers of children with ADHD.
van Rensburg et al. (2018) South Africa (48)	N = 123 (87.8% female); Adults with fibromyalgia (≥18 years old, mean age = 49.9 years)	Fibromyalgia (modified ACR 2010 criteria)	ASRS	Positive screening for adult ADHD was observed in 44.7% of patients with fibromyalgia. Patients with a positive ADHD screen exhibited significantly higher fibromyalgia impact (FIQR scores) and more severe self-reported cognitive impairment compared to those with a negative screen.
Wiwe Lipsker et al. (2018) Sweden (55)	N = 146 (69.9% female); Children and adolescents with chronic pain (8–17 years old, mean age = 14.6 years)	Chronic pain (tertiary clinic sample)	Conners-3 (parent report)	Clinically significant ADHD symptoms were present in 19.9% of children with chronic pain, although only 3.4% had a prior ADHD diagnosis. While pain severity did not differ by ADHD status, pain in children with ADHD symptoms was significantly more likely to be triggered by new or family situations.
Yılmaz & Tamam (2018) Turkey (57)	N = 132 (78 cases/54 controls); Adult women (18–55 years old, mean age = 40.3 years for cases)	Fibromyalgia (2010 ACR criteria)	DSM-5, ASRS, WURS	Adult ADHD was significantly more prevalent in fibromyalgia patients than in controls (29.5% vs. 7.4%), as was childhood ADHD (33.3% vs. 11.1%). Furthermore, fibromyalgia patients exhibited significantly higher levels of impulsivity and ADHD symptoms.
Asztély et al. (2019) Sweden (17)	N = 77 (74 with ADHD); Adult women (19–37 years old, mean age = 27.2 years)	Chronic pain and chronic widespread pain (self-reported)	DSM-IV (childhood diagnosis)	Chronic pain (76.6%) and CWP (32.5%) were common in women with childhood ASD and/or ADHD. Ongoing stimulant treatment in ADHD was associated with a significantly lower prevalence of CWP (16.7% vs. 42.0%).
Yeh et al. (2019) Taiwan (52)	N = 474 (20.3% female); Children and adolescents with ADHD (6–18 years old, mean age = 11.0 years)	Significant pain and pain-induced functional impairment (self-reported)	DSM-IV-TR, SNAP-IV	Significant pain and pain-induced functional impairment were associated with bullying victimization (both physical and verbal/relational). Furthermore, participants with pain and functional impairment experienced more severe depression and anxiety and poorer sleep quality.
Karaş et al. (2020) Turkey (46)	N = 122 (64 cases/58 controls); Adult women (≥18 years old, mean age = 39.8 years for cases)	Fibromyalgia (2010 ACR criteria)	WURS, ASRS	Both childhood and adult ADHD symptom scores were significantly higher in fibromyalgia patients compared to controls. Furthermore, childhood ADHD symptoms predicted fibromyalgia impact (FIQ scores), and this relationship was mediated by depression and anxiety levels.
Kasahara et al. (2020) Japan (25)	N = 153 (62.7% female); Adults with chronic pain and probable somatic symptom disorder (mean age = 54.7 years)	Chronic pain (tertiary pain clinic sample)	DIVA 2.0 (DSM-5), CAARS-S/O, WURS	Adult ADHD was diagnosed in 72.5% (n = 111) of patients with chronic pain and probable somatic symptom disorder, none of whom had a prior ADHD diagnosis. Importantly, these patients exhibited high childhood ADHD symptom scores (WURS), indicating that their ADHD traits had been present yet unrecognized since childhood.
Kabukçu et al. (2021) Turkey (44)	N = 209; Adolescent girls (13–18 years old, mean age = 15.5 years)	Primary dysmenorrhea (self-reported, VAS)	T-DSM-IV-S	Adolescent girls with primary dysmenorrhea affecting their daily activities exhibited significantly higher levels of inattention and hyperactivity-impulsivity compared to those without.
Kasahara et al. (2021) Japan (14)	N = 60 (51.7% female); Adult patients with persistent CNLBP (≥18 years old, mean age = 54.9 years)	Persistent CNLBP	CAARS-S/O	Clinically significant ADHD symptoms (positive on both CAARS-S and CAARS-O) were present in 31.7% of patients. Overall, ADHD indices were significantly higher in patients with persistent CNLBP than in standardized population samples. Hyperactivity/impulsivity scores were positively correlated with both pain intensity and pain duration.
Kindgren et al. (2021) Sweden (69)	N = 201 (56.2% female, 113 with HSD, 88 with hEDS); Children and adolescents with HSD or hEDS (6–18 years old, mean age = 12.0 years)	HSD or hEDS (Pain reported in 78% of the sample) (ICD-10)	ICD-10 (medical record)	The overall prevalence of ADHD in children with HSD or hEDS was 16.0% (approximately three times higher than that in the general population). A higher prevalence of ADHD was observed in children with hEDS than in those with HSD (23% vs. 11%).
Pallanti et al. (2021) Italy (42)	N = 106 (89.6% female); Adults with fibromyalgia (mean age = 41.7 years)	Fibromyalgia (2010 ACR criteria)	DIVA 2.0 (DSM-5)	ADHD was present in 24.5% of patients with fibromyalgia and was associated with greater symptom severity and functional impairment. Patients with both FMS and ADHD exhibited a significantly higher frequency of substance use disorders (38.5% vs. 3.8%), mainly involving opioids.
Türkoğlu & Selvi (2021) Turkey (66)	N = 153 (113 cases/40 controls); Adult women (18–65 years old, mean age = 41.4 years for cases)	Fibromyalgia (2016 ACR criteria)	DSM-5, ASRS	Adult ADHD was significantly more prevalent in patients with fibromyalgia than in controls (38.9% vs. 5.0%). Higher ADHD symptom severity was associated with greater fibromyalgia impact (FIQ) and poorer quality of life. Furthermore, ADHD symptom severity partially mediated the relationship between depression severity and physical quality of life.
Chruciel et al. (2022) USA (47)	N = 3,544,292 (56.4% female); Adults identified from electronic health records (mean age = 50.0 years)	Non-cancer pain (arthritis, musculoskeletal, neuropathies, headache, back/neck pain, fibromyalgia, chronic pain) (ICD-9/10)	Medical records (ICD-9/10)	All non-cancer pain conditions were associated with increased odds of ADHD diagnosis (adjusted ORs = 1.41–1.69).
Ibrahim & Hefny (2022) Egypt (74)	N = 227 (75.8% female); Medical and health sciences students (mean age = 21.9 years)	Chronic back pain (self-reported)	ASRS	Students with chronic back pain exhibited significantly higher ADHD symptom scores (particularly in the attention-deficit domain) and higher central sensitization levels. Furthermore, mediation analysis suggested that central sensitization mediates the effect of ADHD symptoms on chronic back pain.
Stelcer et al. (2022) Poland (58)	N = 240 (90.0% female); Young adults (19–42 years old, mean age = 22.0 years)	Temporomandibular disorders (DC/TMD)	ASRS, DIVA 2.0	Adult ADHD symptoms (positive on ASRS) were identified in 10.0% of the participants. Notably, this ADHD-positive group reported significantly higher temporomandibular pain intensity and experienced greater daytime sleepiness compared to the ADHD-negative group.
Mundal et al. (2023) Norway (38)	N = 263 (40.3% female); Adolescents and young adults with ADHD (13.0–20.5 years old at baseline, mean age = 15.7 years, nine-year follow-up)	Chronic and multisite pain (self-reported)	ICD-10, DSM-IV	The prevalence of chronic and multisite pain was significantly higher in adolescents and young adults with ADHD compared to the age-matched general population at all measurement points over the nine-year follow-up period. This elevated risk for pain was particularly prominent in females.
Berggren et al. (2024) Sweden (56)	N = 857 (50.9% female); Children from a general population birth cohort (10–11 years old)	Frequent and multisite pain (self-reported)	SNAP-IV	Frequent pain was reported by 52.5% of children with symptoms of ADHD combined type, compared to 36.2% of children without these symptoms. Hyperactivity/impulsivity symptoms significantly increased the risk of frequent pain (OR = 2.33), whereas inattention did not. Furthermore, multisite pain was significantly more common among girls with hyperactivity compared to boys with similar symptoms (51.4% vs. 27.9%).
Lundqvist & Kerekes (2024) Sweden (71)	N = 1608 (59.8% female); Upper secondary school students (15–19 years old, mean age = 17.2 years)	Pain frequency and intensity (self-reported)	Self-reported diagnosis	Adolescents with ADHD reported significantly higher pain frequency but not intensity compared to those without psychiatric diagnoses. The presence of coexisting depression and/or anxiety further heightened the association between ADHD and pain frequency. Following headache, back pain was the next most common type of pain among adolescents with ADHD. Notably, common ADHD medications did not have a significant impact on pain experiences.
Brown et al. (2025) USA (35)	N = 116 (65.5% female, 71 completed six-month follow-up); Young adults prescribed opioids for acute pain (19–26 years old, mean age = 22.2 years)	Pain interference (PROMIS)	ASRS Screener	Baseline ADHD symptoms significantly predicted moderate-to-severe pain interference at the six-month follow-up (adjusted OR = 1.52). Furthermore, pain catastrophizing was also a significant predictor of future pain interference in the multivariable model, whereas adverse childhood experiences were not.
Kasahara et al. (2025) Japan (20)	N = 4028 (49.7% female); Adults from a general population internet survey (20–64 years old, median age = 45 years)	Chronic pain (self-reported)	ASRS, AQ	The prevalence of positive ADHD screening was significantly higher in the chronic pain group (13.0%) compared to the non-chronic pain group (8.1%), and it increased with higher pain intensity, reaching 38.3% in those with extreme pain. Furthermore, pathway analysis revealed that ADHD symptoms were significantly associated with chronic pain symptoms and intensity both directly and indirectly (via mental health problems), whereas ASD symptoms were not.
Timm et al. (2025) Germany (70)	N = 245 (127 cases/118 controls); Adults (mean age = 51.0 years for cases)	Chronic pain (ICD-10)	CAARS-S	Patients with chronic pain reported significantly higher cognitive complaints (specifically hyperactivity and motor agitation on the CAARS) and showed objective cognitive impairments compared to healthy controls. Importantly, mediation analysis indicated that the impact of chronic pain on disability was mediated by these subjective cognitive complaints (hyperactivity), emotional factors, and fatigue, but not by objective cognitive impairments.

This table summarizes epidemiological studies examining the prevalence of and associations between attention-deficit/hyperactivity disorder (ADHD) and chronic pain in general population samples and clinical populations. Information is provided on study populations, pain conditions, ADHD assessment methods, and key findings regarding comorbidity rates and associations between ADHD symptoms or diagnoses and pain outcomes.

ADHD, attention-deficit/hyperactivity disorder; ACR, American College of Rheumatology; AQ, Autism-Spectrum Quotient; ASD, autism spectrum disorder; ASRS, Adult ADHD Self-Report Scale; CAARS-S/O, Conners’ Adult ADHD Rating Scales Self-report/Observer-rated; CNLBP, chronic nonspecific low back pain; Conners-3, Conners 3rd Edition; CWP, chronic widespread pain; DC/TMD, Diagnostic Criteria for Temporomandibular Disorders; DIVA, Diagnostic Interview for ADHD in Adults; DSM-IV(-TR)/5, Diagnostic and Statistical Manual of Mental Disorders, Fourth Edition (Text Revision)/Fifth Edition; FIQ, Fibromyalgia Impact Questionnaire; FIQR, Revised Fibromyalgia Impact Questionnaire; hEDS, hypermobile Ehlers–Danlos syndrome; HSD, hypermobility spectrum disorders; ICD-9/10, International Classification of Diseases, Ninth/Tenth Revision; ICHD-3, International Classification of Headache Disorders, 3rd edition; K-SADS-PL, Schedule for Affective Disorders and Schizophrenia for School-Age Children—Present and Lifetime Version; N, number of participants; OR, odds ratio; PROMIS, Patient-Reported Outcomes Measurement Information System; RAP, recurrent abdominal pain; RR, relative risk; SNAP-IV, Swanson, Nolan, and Pelham-IV Questionnaire; T-DSM-IV-S, Turgay Diagnostic and Statistical Manual of Mental Disorders, 4th edition-Based Child and Adolescent Disruptive Behavior Disorders Screening and Rating Scale; TMD, temporomandibular disorder; VAS, visual analogue scale; WURS, Wender Utah Rating Scale.

### 3.1 Characteristics of included studies

The distribution of the included studies by year of publication is shown in **Supplementary Figure 1**. The earliest studies were published by Holmberg and Hjern (45) and Young and Redmond (13). An increase in the number of publications was observed from 2018 onward, with a steady output of studies continuing after 2021.

The geographical distribution of the studies is presented in **Supplementary Figure 2**. Japan (n = 15), Sweden (n = 9), and Turkey (n = 6) accounted for the largest proportion of publications, collectively representing 60.0% of the included studies. Additional studies were conducted in Norway, the United States, Spain, the Netherlands, the United Kingdom, and other countries.

**Figure 2:**
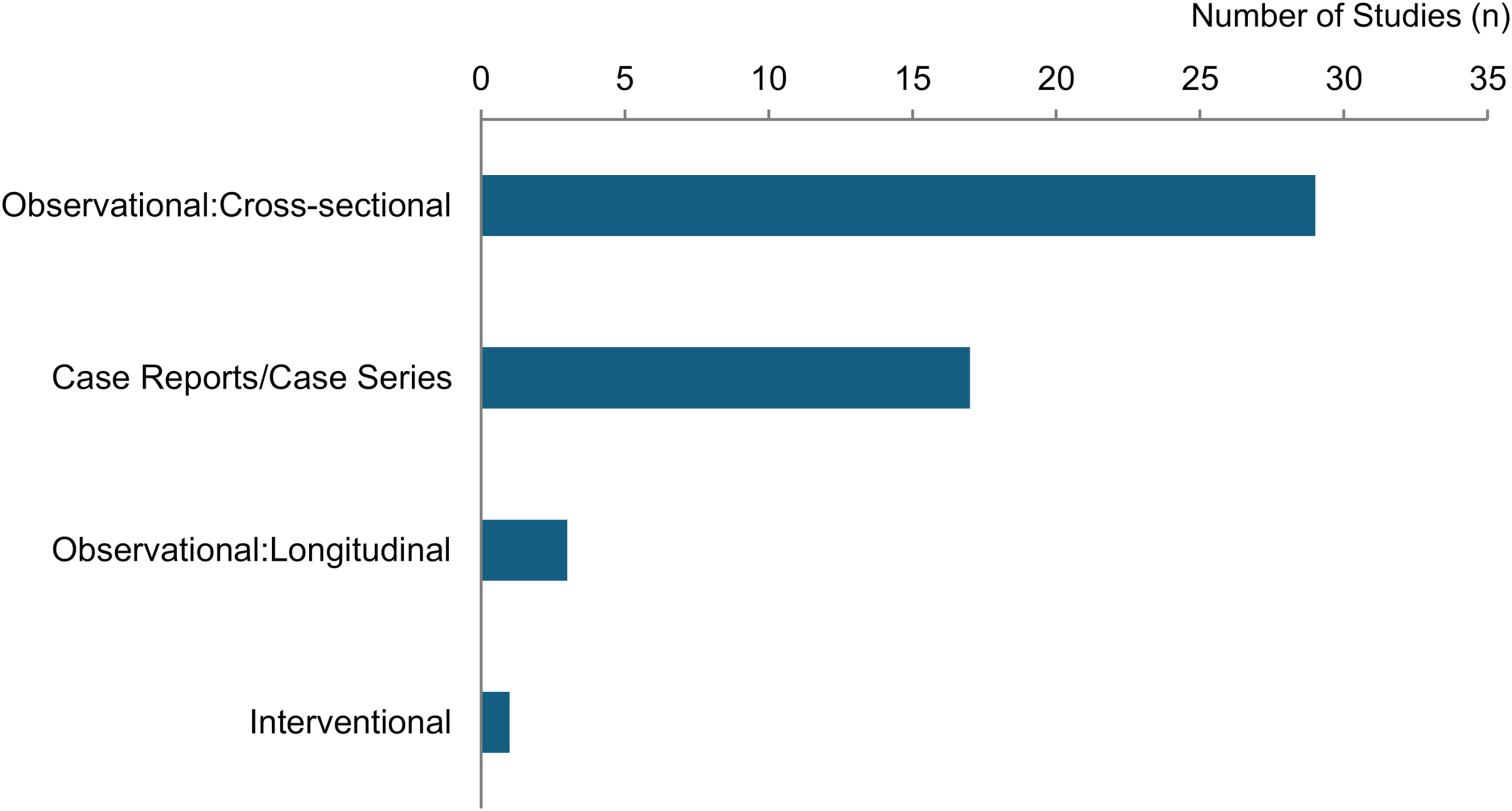
Distribution of study designs. This image categorizes the 50 included studies by study design. Observational cross-sectional studies were the most common (n = 29, 58.0%), followed by case reports and case series (n = 17, 34.0%) and longitudinal observational studies (n = 3, 6.0%). Only one interventional study (2.0%) was identified.

The distribution of the study designs is presented in **Figure 2**. Observational studies constituted the majority of the included studies (n = 32), with cross-sectional designs being the most common (n = 29). These included large population-based surveys and cross-sectional investigations targeting specific pain conditions. Longitudinal studies (n = 3) included prospective cohort and retrospective studies based on medical records.

Case reports and series (n = 17) were also represented. In addition to contemporary clinical cases, these included reinterpretations of historical figures (37, 49). Such publications provided detailed descriptions of individual clinical courses and treatment responses.

Only one interventional study was identified, consisting of a single-arm trial targeting pediatric chronic pain (65).

### 3.2 Epidemiology and prevalence

Of the 50 studies included in this review, 28 (56.0%) focused primarily on the epidemiology and prevalence of ADHD in either general population samples or specific chronic pain conditions. Detailed characteristics and key findings of these studies are presented in **Table 2**.

Across these studies, comorbidities or associations between ADHD and chronic pain were reported in conditions such as fibromyalgia, chronic low back pain, and chronic pain in the general population. In this section, we summarize the findings regarding the association between ADHD and chronic pain in adults and in children/adolescents, as well as the longitudinal risk patterns.

#### 3.2.1 Association and prevalence in adults

Associations between ADHD and chronic pain have been reported in adult populations. The evidence comes from both large-scale epidemiological studies and clinical samples recruited from specialized settings.

#### 3.2.2 Epidemiological associations in the general population

Large epidemiological studies have demonstrated associations between ADHD symptoms and chronic pain. In a cross-sectional study of 7,403 adults in the United Kingdom, Stickley et al. reported that individuals with ADHD symptoms (ASRS score ≥ 14) had significantly higher odds of reporting “extreme pain” compared with those without such symptoms (OR = 3.15) (43). Similarly, in a study of 3,908 Canadian women, Fuller-Thomson et al. found that women with ADHD had a significantly higher prevalence of chronic pain compared with those without ADHD (51).

Using a large U.S. medical records database including more than 3.5 million individuals, Chruciel et al. reported that adults with ADHD had significantly increased odds of receiving non-cancer pain diagnoses, including arthritis, back pain, and fibromyalgia (ORs ranging from 1.4 to 1.7) (47). In Japan, in an internet-based survey of 4,028 participants, Kasahara et al. observed a dose–response relationship between ADHD symptom severity and pain intensity measured using a numerical rating scale (NRS) (20).

#### 3.2.3 Comorbidity in clinical populations

Higher comorbidity rates have been reported in clinical samples of patients presenting to specialized pain clinics. In a study of 153 patients with treatment-resistant chronic pain at a university hospital pain center, Kasahara et al. found that 72.5% met the diagnostic criteria for adult ADHD (25). However, it should be noted that this cohort comprised patients with suspected somatic symptom disorder and refractory pain, and thus may have been subject to selection bias.

Similarly, Derksen et al. reported that adult ADHD was diagnosed in 25% of patients with fibromyalgia (67). In addition, van Rensburg et al. found that 44.7% of patients with fibromyalgia screened positive for adult ADHD symptoms and that ADHD-positive individuals showed greater disease impact and cognitive dysfunction (48).

Stray et al. further reported frequent widespread pain and motor regulation abnormalities among adults with ADHD, suggesting potential links between ADHD-related characteristics and pain experiences (53).

Collectively, these findings suggest that the association between ADHD and chronic pain may be clinically meaningful across different adult clinical populations.

#### 3.2.4 Association and prevalence in children and adolescents

Associations between ADHD and chronic pain have also been reported in pediatric and adolescent populations. Evidence is available from both population-based studies and clinical samples.

#### 3.2.5 Epidemiological associations in the general population

Large studies of children in the general population have identified associations between ADHD symptoms and pain-related complaints. In a Swedish cohort study of 10-year-old children, Holmberg and Hjern reported that children with ADHD had approximately 2.2 times higher relative risk of recurrent abdominal pain (RAP) compared with those without ADHD (45). Increased rates of sleep disturbance and fatigue were also observed.

In a study of children aged 10–11 years, Berggren et al. found that 52.5% of children with ADHD symptoms—particularly hyperactivity/impulsivity—reported pain at least once per week, compared with 36.2% in the control group (56). These findings suggest that ADHD traits may be associated with increased pain experiences, even in the absence of a formal diagnosis.

#### 3.2.6 Comorbidity in clinical samples

Studies of pediatric patients in specialized clinics have reported higher comorbidity rates. These findings can be broadly categorized into two perspectives: (1) the detection of ADHD symptoms in pediatric pain clinics and (2) the prevalence of chronic pain in pediatric ADHD or psychiatric clinics.

ADHD symptoms in pediatric pain clinics: Wiwe Lipsker et al. reported that 19.9% of children and adolescents attending a tertiary pain clinic exhibited clinically significant ADHD symptoms based on the Conners-3 assessment (55). When comorbid autism spectrum disorder (ASD) was included, the proportion increased to 26%. Notably, many patients had not received a prior diagnosis of a neurodevelopmental disorder.

Chronic pain in ADHD or psychiatric clinics: Mangerud et al. reported that 65.9% of adolescents with ADHD attending a psychiatric outpatient clinic experienced pain lasting longer than three months (68). Similarly, Kutuk et al. found that children diagnosed with ADHD had significantly higher prevalence rates of migraine and RAP than controls (39).

In addition, a 9-year longitudinal study by Mundal et al., based on the Trøndelag Health Study (HUNT) cohort (38), indicated that adolescents with ADHD, particularly female adolescents, were more likely to report chronic pain in young adulthood. Among women in their 20s with ADHD, 75.9% reported chronic pain (38). However, this estimate was derived from a specific subgroup and should be interpreted with caution.

Overall, these findings suggest that the association between ADHD and chronic pain may persist from childhood through adolescence and into early adulthood.

#### 3.2.7 ADHD as a potential risk factor for pain chronicity

ADHD symptoms may not only co-occur with chronic pain but also be associated with an increased risk of future chronic pain and pain-related functional impairment.

The prospective longitudinal study by Asztély et al. is a key study examining long-term risk (17). In this study, female participants diagnosed with ADHD during childhood were followed up to 16–19 years later. In adulthood, 76.6% reported chronic pain, and 32.5% met the criteria for chronic widespread pain (CWP). These proportions were higher than those typically reported in the general population, suggesting a potential association between childhood ADHD and an increased risk of chronic pain in adulthood. However, interpretations should be made with caution, as the study sample was limited to female participants.

Regarding the transition from acute to chronic pain, Brown et al. conducted a prospective cohort study of young adults who sought medical care for acute pain (35). Baseline ADHD symptoms significantly predicted pain interference at 6-month follow-up (OR = 1.52). Although depressive symptoms and pain catastrophizing were also associated with pain outcomes, ADHD symptoms remained an independent predictor in multivariable analyses.

Taken together, these longitudinal findings suggest that ADHD traits may be associated with the development of chronic pain and the persistence of pain-related functional impairment. However, further research is required to clarify the causal pathways and potential mediating mechanisms.

### 3.3 Assessment methods of ADHD

**Figure 3:**
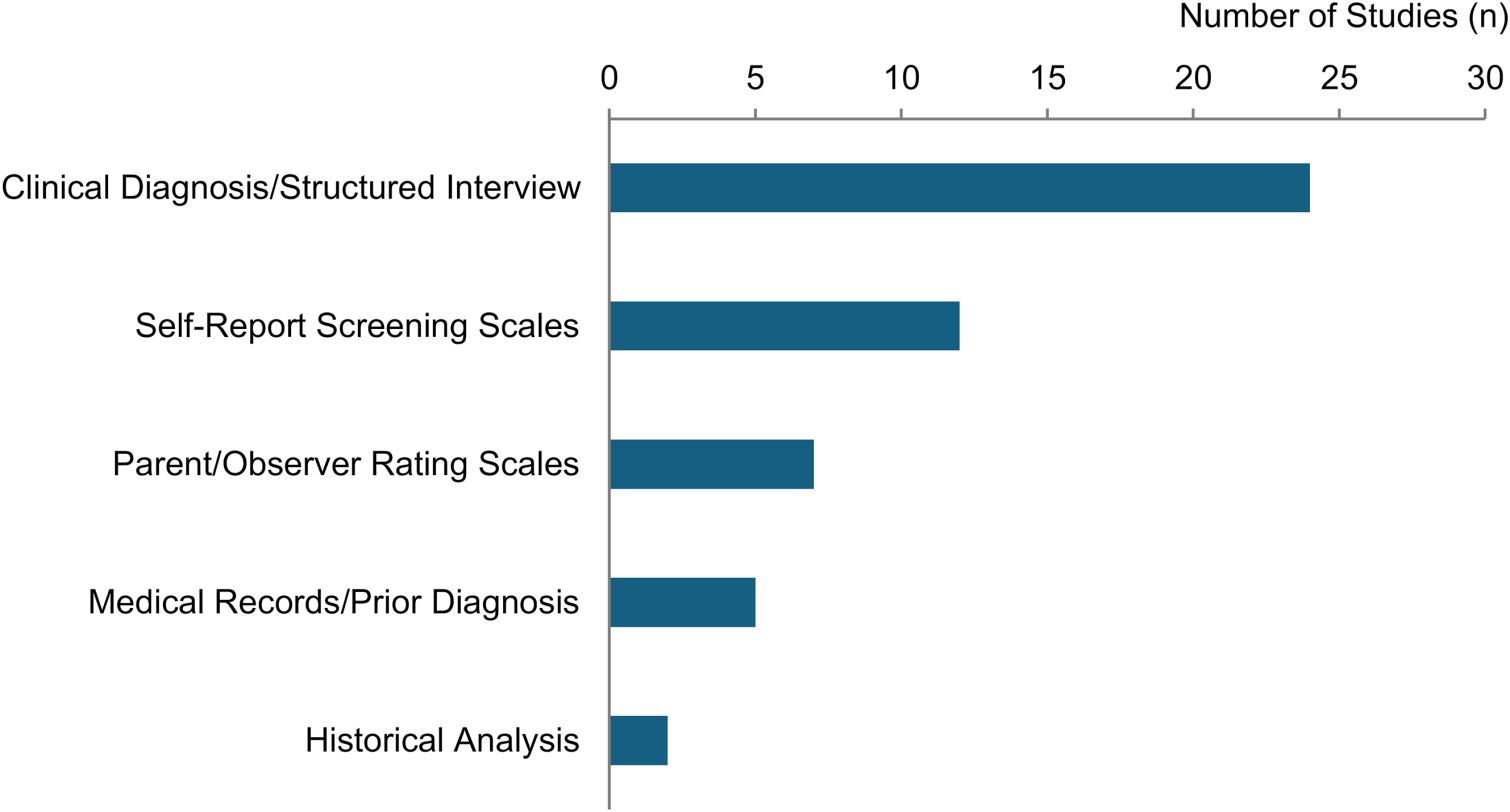
Distribution of ADHD assessment methods. This image classifies the methods used to assess ADHD in the 50 included studies. Clinical diagnosis or structured interviews (e.g., DIVA, K-SADS) were used in 24 studies (48.0%), self-report screening scales in 12 studies (24.0%), parent or observer rating scales in 7 studies (14.0%), medical records or previously documented diagnoses in 5 studies (10.0%), and historical analyses in 2 studies (4.0%). ADHD, attention-deficit/hyperactivity disorder; DIVA, Diagnostic Interview for ADHD in Adults; K-SADS, Kiddie Schedule for Affective Disorders and Schizophrenia.

The methods used to assess ADHD in the included studies varied considerably, ranging from reliance on self-reported screening instruments to comprehensive diagnostic evaluations conducted by trained clinicians using structured interviews. The distribution of ADHD assessment methods across the included studies is shown in **Figure 3**.

In this review, the studies were hierarchically categorized according to the most rigorous diagnostic methods employed.

#### 3.3.1 Clinical diagnosis and structured interviews

In 24 studies (48.0%), ADHD was defined based on clinician-confirmed diagnoses according to the DSM-IV (75), DSM-5 (10), or ICD-10 (76) criteria, or through structured diagnostic interviews, such as the Diagnostic Interview for ADHD in Adults (DIVA 2.0), Kiddie Schedule for Affective Disorders and Schizophrenia (K-SADS), or Structured Clinical Interview for DSM (SCID). These included both clinical research and case reports.

In more recent studies, multimodal assessment approaches were increasingly observed, combining structured diagnostic interviews with validated rating scales (e.g., ASRS) to assess symptom severity.

#### 3.3.2 Self-reported and observer-reported screening scales

In 12 studies (24.0%), ADHD was defined solely based on validated screening instruments without the use of structured diagnostic interviews.

Commonly used instruments included the ASRS in adult populations and the Conners’ Rating Scales and SNAP-IV in pediatric samples. The WURS was used in some adult studies for retrospective assessment of childhood ADHD symptoms.

#### 3.3.3 Medical records and registry-based definitions

In five studies (10.0%), ADHD was identified using diagnostic codes (ICD-9 or ICD-10) (76, 77) or prescription records extracted from medical or registry databases.

Although these studies benefited from large sample sizes, detailed information regarding individual diagnostic procedures was not available.

### 3.4 Clinical phenotypes of comorbid pain

Comorbidity between ADHD and chronic pain was reported across a wide spectrum of clinical phenotypes, ranging from generalized pain syndromes to chronic pain localized to specific anatomical regions.

**Figure 4:**
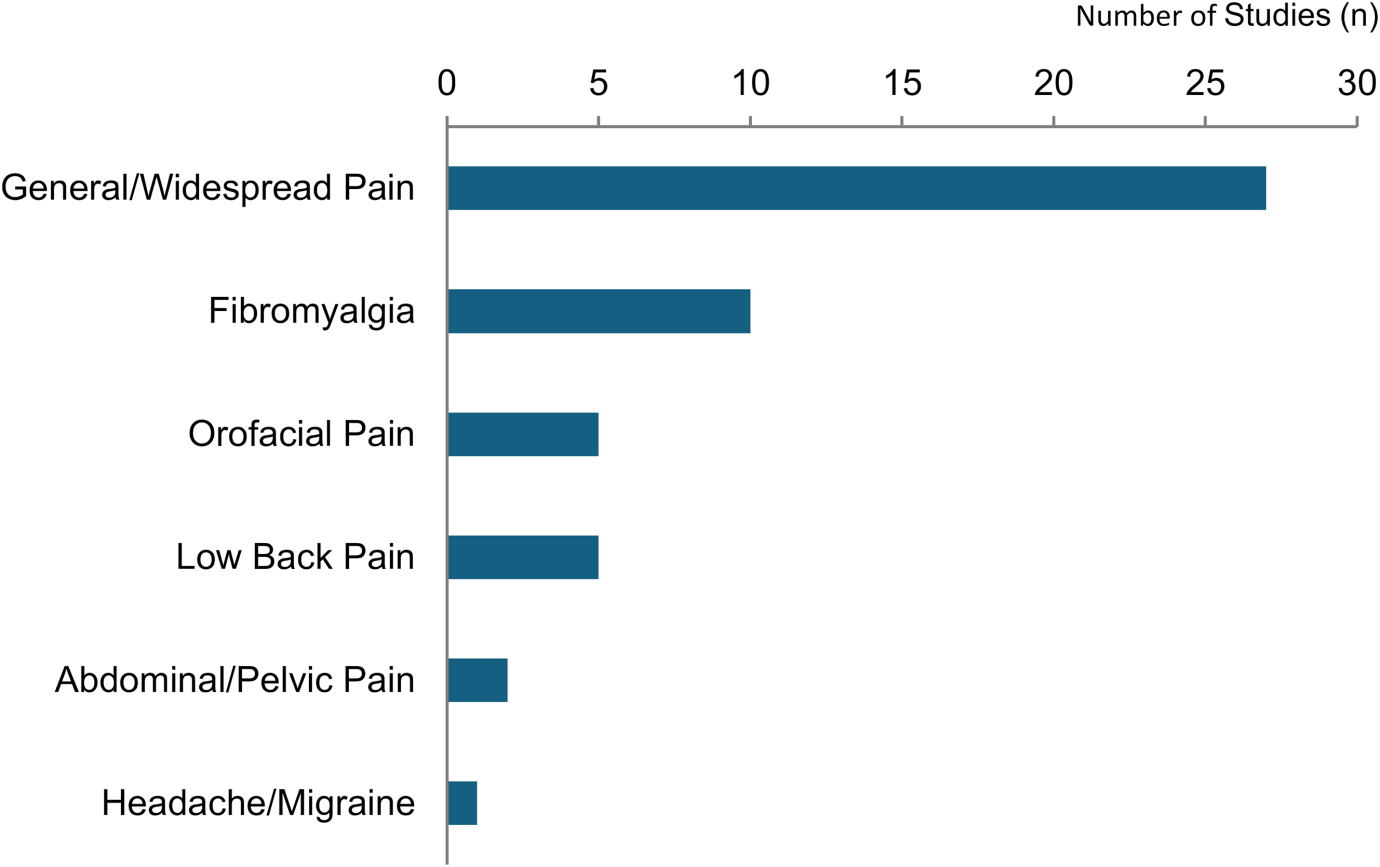
Distribution of research focus by pain phenotype. This image categorizes the 50 included studies according to pain phenotype. General or widespread pain (n = 27, 54.0%) and fibromyalgia (n = 10, 20.0%) accounted for the majority. Additional studies focused on site-specific pain conditions, including orofacial pain (n = 5, 10.0%), low back pain (n = 5, 10.0%), abdominal or pelvic pain (n = 2, 4.0%), and migraine or headache (n = 1, 2.0%).

**Figure 4** shows the distribution of the included studies according to the pain site or diagnostic category identified in this review.

#### 3.4.1 Fibromyalgia and Other Widespread Pain Conditions

Twenty-seven studies (54.0%) were classified within this category.

Fibromyalgia: Fibromyalgia was addressed in ten studies. Across these studies, reported rates of ADHD comorbidity ranged from 24% to 45%, which exceed estimated prevalence rates in the general population.

Using structured diagnostic interviews, Derksen et al. reported that 25% of patients with fibromyalgia met the diagnostic criteria for adult ADHD (67). Similarly, van Rensburg et al. found an ASRS-positive rate of 44.72% among individuals with fibromyalgia and reported that the comorbid group demonstrated higher cognitive dysfunction and greater disease severity scores (48).

Other studies, including those by Reyero et al., Pallanti et al., and Yılmaz and Tamam, reported adult ADHD comorbidity rates ranging from 24% to 32% in fibromyalgia populations (40, 42, 57). In a case report, Young suggested that undiagnosed ADHD may be present in some patients with fibromyalgia or chronic fatigue syndrome (13).

Other chronic or widespread pain conditions: 27 studies examined chronic or widespread pain without specifying a particular diagnostic category. These included both large epidemiological investigations and studies on patients attending specialized clinics.

In addition, the physical factors associated with generalized pain were explored in certain studies. For example, among children with hypermobility spectrum disorders or hypermobile Ehlers–Danlos syndrome (HSD/hEDS), an ADHD comorbidity rate of 16% and pain prevalence of 78% were reported, suggesting that connective tissue characteristics may contribute to shared vulnerability to both pain and ADHD (69).

#### 3.4.2 Site-specific pain

Several studies reported ADHD comorbidity in chronic pain localized to specific anatomical regions.

Orofacial pain: Five studies focused on orofacial pain, including atypical odontalgia (60), burning mouth syndrome (63), and persistent idiopathic facial pain (28).

Studies involving treatment-resistant cases reported that a proportion of patients met diagnostic criteria for ADHD (16, 63). Additionally, some reports have described improvements in pain intensity and catastrophizing scores following treatment with ADHD medications (16, 28, 60, 61, 63). However, these findings are largely derived from case reports or small clinical samples.

Chronic low back pain: Five studies examined chronic low back pain. These studies reported that a subset of patients with chronic low back pain without clear structural abnormalities exhibited clinically significant ADHD symptoms (14).

Migraine and other pain conditions: Studies investigating migraines (39), abdominal pain (62), and dysmenorrhea (44) were also included. Both pediatric/adolescent and large administrative database studies have reported associations between ADHD and various non-cancer pain diagnoses (47).

### 3.5 Pathophysiological mechanisms

The remaining 22 studies reported on pathophysiological mechanisms, potential biomarkers, and treatment-related outcomes in individuals with comorbid ADHD and chronic pain. An overview of these studies is provided in **Table 1**. These investigations included neurochemical hypotheses, neuroimaging studies, research on motor and sensory function, and observational studies and case reports examining the effects of ADHD medications.

To facilitate conceptual synthesis, the mechanisms proposed in the included studies were categorized according to a three-system framework comprising motor regulation, sensory-attentional processing, and descending pain modulation, with additional categories for brain network alterations and neuroimmune mechanisms.

A potentially shared neurobiological basis underlying the comorbidity of ADHD and chronic pain has been discussed in the literature. In this section, findings related to brain function as well as motor, sensory, and immunological factors are summarized.

#### 3.5.1 Brain function and neuroimaging

Both ADHD and chronic pain have been linked to alterations in dopaminergic and noradrenergic systems (18, 19). Given reports of a high prevalence of ADHD symptoms among individuals with chronic pain (20, 25), overlapping neural networks involved in pain modulation and attentional control have been hypothesized.

In a retrospective study using cerebral blood flow single-photon emission computed tomography (SPECT), Takahashi et al. examined 65 patients with nociplastic pain comorbid with ADHD (23). Increased regional cerebral blood flow was observed in the precuneus, insular cortex, and thalamus before treatment. The precuneus, a core region of the DMN, showed a positive correlation between cerebral blood flow values and clinical severity (Clinical Global Impression–Severity Scale, CGI-S). Following treatment with methylphenidate, reductions in precuneus blood flow and improvements in pain symptoms were reported. These findings suggest the involvement of pain-related brain networks in patients with comorbid ADHD, although causal relationships cannot be established.

#### 3.5.2 Motor, sensory, and immunological factors

Muscle tone, motor regulation, and connective tissue characteristics: Stray et al. reported a high prevalence of impaired motor inhibition and increased muscle tone in adults with ADHD (53). In this study, many individuals with ADHD reported widespread pain, and pain intensity was associated with motor regulation indices.

Similarly, in a study focused on 121 psychiatric outpatients, Udal et al. found that individuals with ADHD reported significantly higher rates of trunk and widespread pain than those without ADHD, and these pain reports were associated with measures of muscle tone (54).

In addition, children with HSD/hEDS have been reported to exhibit high rates of both ADHD and pain comorbidity (69). Although these findings suggest a possible link between neurodevelopmental traits and connective tissue characteristics, the underlying mechanisms remain unclear.

Collectively, these studies suggest a potential association between motor regulation abnormalities and chronic pain in individuals with ADHD.

Sensory hypersensitivity: Lipsker et al. reported that children with chronic pain who exhibited ADHD or ASD traits showed higher levels of sensory over-responsivity (SOR) (55).

In a study of adolescents and young adults with neurodevelopmental disorders (ASD and ADHD), Martínez-González et al. found that sensory hypersensitivity was associated with greater severity of pain and gastrointestinal symptoms (36).

These findings suggest that sensory processing characteristics may be linked to pain experiences in individuals with neurodevelopmental conditions.

A cross-sectional study of medical students indicated an association between ADHD symptoms and chronic back pain; however, this association became non-significant after adjusting for central sensitization (74). This finding suggests that central sensitization may mediate the relationship between ADHD traits and chronic back pain.

Neuroimmunological factors: Endres et al. reported the case of a 36-year-old woman presenting with ADHD-like symptoms and severe pain in whom anti-thalamic antibodies were detected in the cerebrospinal fluid (41). The patient also had elevated dopamine levels and reduced glutamate and serotonin levels. Temporary symptom improvement was observed after steroid treatment.

Although limited to a single case report, this observation raises the possibility that neuroimmunological mechanisms may contribute to symptom presentation in a subset of individuals with comorbid ADHD and chronic pain.

### 3.6 Therapeutic interventions and outcomes

This section summarizes the reports on therapeutic interventions in individuals with comorbid ADHD and chronic pain. Interventions included pharmacological treatments (including off-label use of ADHD medications) as well as psychosocial and non-pharmacological approaches. Most reports on treatment effects were derived from case reports or non-randomized studies, and RCTs were extremely limited.

#### 3.6.1 Pharmacological interventions: ADHD medications and pain outcomes

The distribution of therapeutic intervention studies is illustrated in **Supplementary Figure 3**. Pharmacological treatments accounted for the majority of reports.

Psychostimulants (n = 15) were the most frequently reported agents, followed by atomoxetine (n = 10), antipsychotic medications with dopaminergic partial agonist properties (sometimes referred to as dopamine system stabilizers) (n = 6), and α2-adrenergic receptor agonists (n = 6). Several studies reported reductions in pain scores following administration of ADHD medications (62, 63). However, most of these reports were based on case studies or small observational samples.

Historical reinterpretation: As a historical reinterpretation, Kasahara et al. conducted a literature-based review of John F. Kennedy’s chronic low back pain (37). The authors discussed a possible association between amphetamine use and the clinical course of pain; however, the analysis was hypothesis-generating and exploratory in nature.

Several case reports and small-scale studies have described the use of ADHD medications in patients with complex, comorbid medical and psychiatric conditions. For example, a case report described substantial improvement in fibromyalgia pain following treatment of ADHD with atomoxetine (73). Additionally, in an adolescent patient with chronic low back pain associated with autosomal dominant polycystic kidney disease (ADPKD), a reduction in pain scores was reported following combined treatment with guanfacine and methylphenidate (64). Changes in cerebral blood flow on SPECT imaging were also reported; nonetheless, causal relationships cannot be established based on this single case.

Zain et al. described an adult patient with longstanding, multisite chronic pain, including chest and back pain, who had been refractory to multiple treatments, including antidepressants, epidural blocks, and even electroconvulsive therapy (ECT) (26). Undiagnosed ADHD was subsequently identified, and after initiation of osmotic-release oral system methylphenidate (OROS-MPH), the patient’s pain intensity (NRS) decreased from 6 to 0 with complete resolution (26). Although notable, this observation is based on a single case and should be interpreted with caution.

Additional reports included a case of chronic pain comorbid with post-traumatic stress disorder, in which high-dose atomoxetine was associated with improvements in both pain and psychiatric symptoms (27), and a case of abdominal pain comorbid with depression, in which combined treatment with methylphenidate and a serotonin–norepinephrine reuptake inhibitor (SNRI) was associated with improvements in pain and functional outcomes (62).

Overall, while these reports suggest a possible association between ADHD-targeted pharmacotherapy and improvements in pain outcomes, the evidence is primarily derived from uncontrolled designs.

#### 3.6.2 Psychological and non-pharmacological approaches

In addition to pharmacological interventions, several studies have examined psychological approaches to support individuals with comorbid ADHD and chronic pain. In particular, acceptance and commitment therapy (ACT) and related behavioral interventions have been reported.

ACT: Balter et al. conducted an ACT-based intervention study focused on pediatric patients with chronic pain (65). Improvements were observed in pain interference, psychological flexibility, and functional impairment, regardless of the level of ADHD symptoms or autistic traits. Notably, children with higher levels of autistic traits demonstrated greater improvements in insomnia and emotional functioning. These findings suggest that ACT-based interventions may be applicable across neurodevelopmental profiles, although the study design limits causal inferences.

Parental behavioral training: Psychological interventions may also target family systems in addition to the affected child. In a case report, Wiwe Lipsker et al. described a six-year-old girl with severe chronic pain comorbid with ASD and ADHD, who received methylphenidate in combination with a ten-session ACT-based parent training program (50). Following the intervention, reductions in parental depressive symptoms and pain-related overprotective behaviors were reported. Concurrently, substantial reductions were observed in the child’s pain and functional impairment, including an improvement in return to school attendance. However, as this report was based on a single case, its generalizability is limited.

### 3.7 Psychosocial impact and associated factors

The comorbidity between ADHD and chronic pain has been reported to exert multifaceted effects on functional status and psychosocial domains (17, 59). In a cross-sectional study of patients with chronic pain, Timm et al. reported an association between ADHD-related symptoms—particularly hyperactivity and motor restlessness—and pain-related disability. Furthermore, they found that ADHD symptoms partially mediated the relationship between pain intensity and pain-related disability (70). Previous studies have primarily examined this relationship from three perspectives: (1) QOL and pain interference, (2) interaction with mental health, and (3) environmental and social stressors (35, 51, 52).

QOL and pain interference: Among individuals with chronic pain who exhibit ADHD traits, reduced health-related quality of life and increased pain interference have consistently been reported (17, 59, 66). Studies involving adult women, pediatric patients with chronic pain, and young adults have demonstrated significant associations between ADHD symptoms and lower QOL or greater functional impairment (17, 51, 59). Longitudinal data further suggest that ADHD symptoms may predict future pain interference (35).

Interaction with mental health: The coexistence of ADHD and chronic pain has been associated with increased rates of depression, anxiety, sleep disturbance, and suicidal ideation, implicating greater clinical complexity (46, 51, 52). In particular, pain interference has been shown to be independently associated not only with ADHD symptoms, but also with psychological factors such as pain catastrophizing (35), highlighting the multifactorial nature of symptom burden.

Environmental and social stressors: Social stressors, including bullying and adverse childhood experiences (ACEs), have been suggested as potential risk factors for pain and functional impairment in individuals with ADHD (45, 52). Early life adversity has also been associated with increased vulnerability to both chronic pain and later mental health difficulties (51). These findings indicate that psychosocial context may play an important role in shaping the trajectory of comorbid ADHD and chronic pain.

## 4 Discussion

### 4.1 Overlooked comorbidity: from widespread to site-specific pain

This scoping review shows that the comorbidity between ADHD and chronic pain has been reported across multiple countries, cultural contexts, and age groups. Epidemiological studies in the general population have consistently identified significant associations between ADHD symptoms and both chronic pain (20, 56) and pain-related functional impairment (43). Some studies have suggested that greater ADHD symptom burden may be associated with more frequent or more severe pain (20, 43, 56).

Despite these epidemiological findings, the systematic assessment of neurodevelopmental conditions is not routinely integrated into somatic medical care. In particular, outside psychiatric settings, ADHD screening may not be incorporated into standard clinical protocols for patients presenting with chronic pain (25).

Historically, the association between ADHD and pain has been primarily examined in the context of fibromyalgia and chronic widespread pain. Several studies have reported that the proportion of patients with fibromyalgia meeting the diagnostic criteria for adult ADHD exceeds estimates from the general population, or that fibromyalgia is associated with elevated ADHD symptom levels (40, 42, 57, 67). A possible neurobiological overlap between the two conditions has been discussed, particularly involving dopaminergic and noradrenergic system function and sensory processing characteristics (13, 66). However, empirical evidence clarifying causal pathways remains limited.

Notably, the findings synthesized in this review indicate that ADHD comorbidity is not confined to generalized pain syndromes. Associations have also been reported in site-specific and often treatment-resistant conditions, including chronic low back pain (14), orofacial pain (16), and migraine (39). These conditions are typically managed in orthopedic, dental, neurological, or primary care settings. Therefore, underlying neurodevelopmental traits may remain unrecognized in routine clinical practice (24, 25).

Some studies conducted in specialized referral centers have reported relatively high proportions of patients meeting ADHD diagnostic criteria (13, 67). However, these samples frequently comprised treatment-resistant patients referred to tertiary care institutions, and thus referral bias cannot be excluded (16, 39). Therefore, these prevalence estimates should not be directly extrapolated to the general population.

From a clinical perspective, a subset of individuals with chronic pain has been described as exhibiting characteristics such as difficulty sustaining attention (13, 67), restlessness and impulsivity (57, 67), and emotional dysregulation (46, 67). This observation is consistent with previous findings indicating that ADHD-related symptoms, particularly hyperactivity or motor restlessness, may be associated with pain-related disability in patients with chronic pain. In this review, these features were conceptually summarized as potential clinical indicators suggestive of ADHD comorbidity in populations with chronic pain (78) (**Table 3**). However, these characteristics are not specific to ADHD and may also occur in other psychiatric or medical conditions. Therefore, diagnostic confirmation requires structured interviews and/or validated assessment instruments.

**Table 3:** Clinical observational points suggestive of ADHD-related traits in patients with chronic pain.

Domain	Example clinical observations	Clinical considerations (Hypothetical interpretation)
Attention regulation	Frequent topic shifts; incomplete questionnaire responses	May prompt consideration of attentional dysregulation
Hyperactivity/Impulsivity	Excessive talkativeness; intolerance of waiting; observable restlessness	May suggest hyperactive or impulsive traits
Emotional dysregulation	Marked irritability or intense anger in response to pain or clinical interactions	Possible interaction between emotional regulation difficulties and pain experience

This table outlines clinical observations that may prompt the consideration of ADHD-related traits in patients presenting with persistent or treatment-resistant chronic pain. The listed items are conceptually derived from the findings of this scoping review and focus on core ADHD symptoms (inattention; hyperactivity–impulsivity) as well as clinically relevant aspects of emotional dysregulation. Although emotional dysregulation is not included in the formal DSM diagnostic criteria, it has been widely reported as a clinically significant feature of ADHD (78). This table does not represent diagnostic criteria and should not be used as a standalone screening tool. A definitive diagnosis requires a comprehensive psychiatric evaluation, including structured interviews conducted by qualified professionals.

Collectively, these findings suggest that incorporating assessments of neurodevelopmental traits into the evaluation of chronic pain may provide clinically relevant insights. Nevertheless, the feasibility, optimal implementation, and clinical utility of ADHD screening in chronic pain settings require further investigation through prospective studies.

However, substantial heterogeneity was observed across the included studies in terms of the study design, ADHD assessment methods, and definitions of pain. ADHD was assessed using various approaches, including screening instruments and structured diagnostic interviews as well as registry-based diagnoses. Similarly, pain outcomes were defined according to clinical diagnoses, self-reported measures, and scale-based assessments. These variations should be considered while interpreting the current findings.

### 4.2 Hypothetical integrative model: a three-system framework

**Figure 5:**
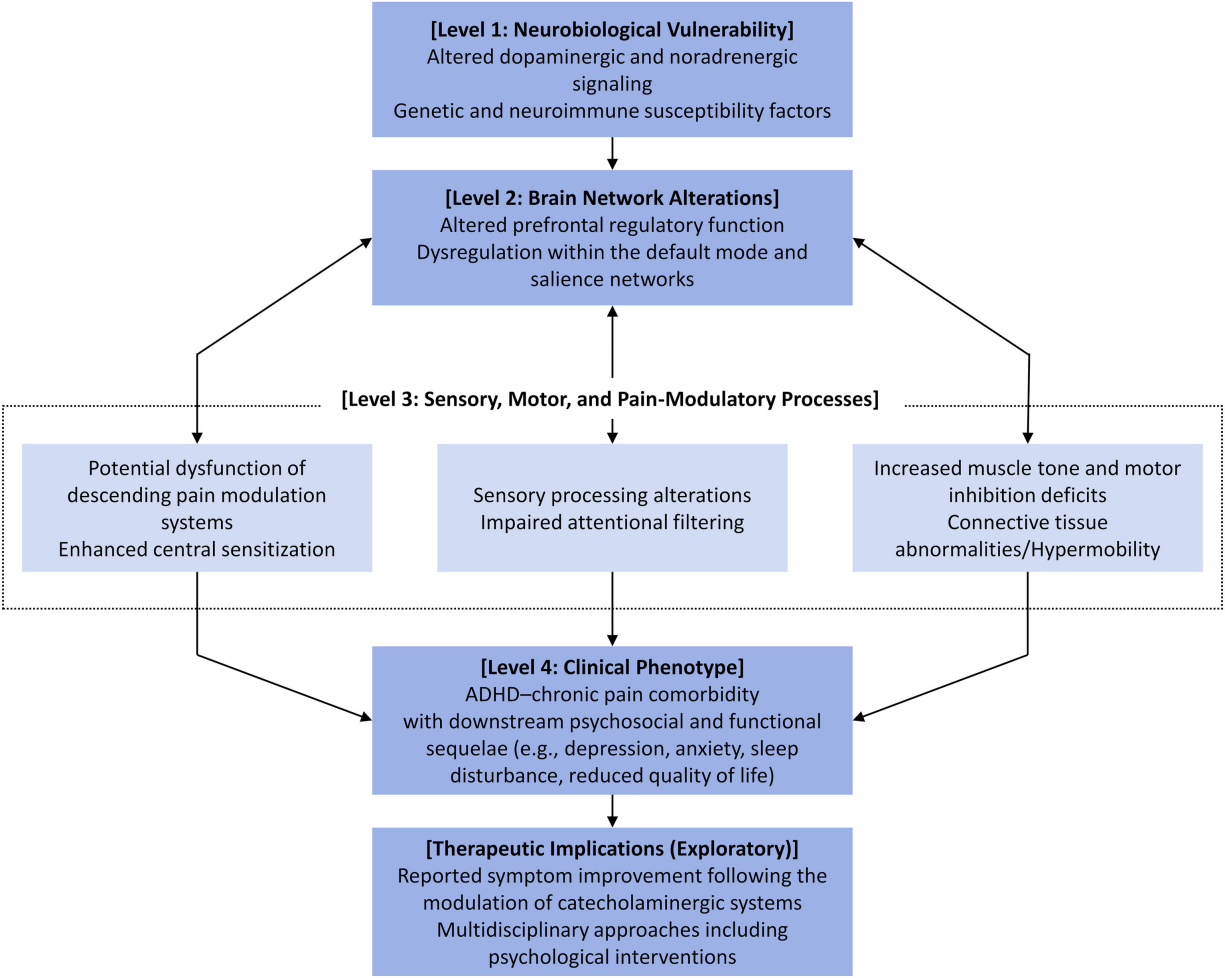
Hypothetical integrative model of ADHD–chronic pain comorbidity. This image presents a hypothetical hierarchical model integrating epidemiological, clinical, and neuroimaging findings to explain the comorbidity between ADHD and chronic pain. ● Level 1 (Neurobiological Vulnerability): Genetic factors, neuroimmunological processes, and dysregulation of the dopaminergic and noradrenergic systems are proposed as foundational mechanisms. ● Level 2 (Brain Network Alterations): These vulnerabilities may be associated with altered prefrontal regulatory function and functional changes within the default mode network and salience network. Neuroimaging studies have reported perfusion changes in regions such as the precuneus and insular cortex. ● Level 3 (Sensory, Motor, and Pain-Modulatory Processes): Network-level alterations may manifest as potential dysfunction of descending pain modulation systems, enhanced central sensitization, altered sensory processing, impaired attentional filtering, increased muscle tone, motor inhibition deficits, and connective tissue abnormalities/hypermobility. ● Level 4 (Clinical Phenotype): These processes may ultimately present as comorbid ADHD symptoms and chronic pain, accompanied by functional impairment (e.g., depression, anxiety, insomnia, reduced quality of life). Therapeutic Implications (Exploratory): Pharmacological treatments targeting catecholaminergic systems and psychosocial interventions have been reported to improve symptoms in some cases. However, these findings are primarily based on observational studies and case reports, and further controlled research is required. ADHD, attention-deficit/hyperactivity disorder.

Integrating the epidemiological, clinical, and neuroimaging findings summarized in this review, the comorbidity between ADHD and chronic pain may be conceptualized as arising from interactions among multiple neurophysiological systems. Based on previously published findings, we propose a hypothetical integrative model to conceptualize the relationship comprising three interacting functional systems (**Figure 5**).

In this framework, neurochemical characteristics are positioned as a foundational layer (Level 1); influencing large-scale brain network function (Level 2); which in turn affects sensory, motor, and pain-modulatory regulatory processes (Level 3); ultimately contributing to the clinical phenotype of chronic pain and functional impairment (Level 4). This model represents a conceptual synthesis of the existing evidence rather than a causal framework. In this model, the associations between ADHD and pain-related outcomes are based on observational findings, and the proposed neurophysiological pathways are hypothetical and intended to generate testable mechanisms.

#### 4.2.1 Motor inhibition and muscle tone regulation

Motor inhibition characteristics have been reported in ADHD, and some studies have suggested associations of ADHD with increased muscle activity or altered muscle tone (53). Associations between trunk or widespread pain and motor regulation indices have also been described (53, 54), although these findings are primarily derived from observational studies.

Additionally, children with HSD/hEDS have been reported to exhibit a relatively high prevalence of ADHD (approximately 16%) and frequent comorbid pain (69). These findings suggest a possible relationship between neurodevelopmental traits and connective tissue characteristics. However, the underlying mechanisms remain unclear.

In chronic musculoskeletal pain, persistent muscle activity has been suggested as a potential contributor to ongoing nociceptive input (53, 54). From a theoretical perspective, motor regulation characteristics could plausibly influence pain experiences. Nevertheless, whether altered muscle tone is the primary contributor to pain or a secondary consequence of chronic pain remains unclear.

#### 4.2.2 Sensory and attentional regulation

ADHD has been associated with alterations in attentional control and sensory processing characteristics (13, 36). In chronic pain research, hypervigilance to pain and sustained attentional focus on somatic sensations have been implicated in symptom persistence (25).

Biases in sensory gating and attentional regulation may theoretically influence pain perception (13, 16), and some studies have reported associations between sensory hypersensitivity or altered sensory responsiveness and greater pain severity and interference (36, 55).

Neuroimaging findings have further demonstrated alterations in activity within default mode network (DMN)-related regions, including the precuneus (23). These observations raise the possibility that altered regulation of internally directed attention may contribute to pain experience. However, the direct causal pathways have not yet been established.

#### 4.2.3 Descending pain modulation and central sensitization

The dopaminergic and noradrenergic systems contribute to the regulation of descending pain inhibitory pathways (18, 21, 22). Given the established involvement of catecholaminergic systems in ADHD (19), it is theoretically plausible that the neurochemical characteristics associated with ADHD may influence pain modulation mechanisms.

In chronic pain research, impaired descending inhibitory function has been associated with central sensitization (22). Although increased pain burden has been reported in individuals with ADHD (20, 43), definitive evidence demonstrating direct dysfunction of the descending inhibitory systems in this population remains limited.

Some neuroimaging studies have reported alterations in cerebral blood flow in regions implicated in pain processing, such as the insular cortex and thalamus (23). Although these findings suggest the involvement of pain-related neural networks, they do not directly establish impaired descending inhibition.

#### 4.2.4 Synthesis

Taken together, the available evidence may be conceptualized within a hypothetical framework involving interactions among three functional systems:

- Increased or sustained nociceptive input potentially related to motor regulation characteristics
- Amplification of pain perception associated with sensory and attentional regulation traits
- Altered pain modulation related to descending inhibitory system characteristics

Interactions among these systems may contribute to the persistence of chronic pain and associated functional impairment.

The proposed model represents a conceptual integration of existing findings. Future validation through prospective longitudinal studies, neurophysiological assessments, and functional neuroimaging investigations is necessary to clarify the causal mechanisms and test the proposed framework.

### 4.3 Clinical implications and future directions

The available evidence varies substantially in methodological rigor, ranging from case reports and small observational studies to large epidemiological analyses. In particular, evidence regarding ADHD-targeted treatment effects in chronic pain is largely derived from uncontrolled studies. Thus, the findings of these studies should be interpreted with caution.

The findings of this review suggest that, in a subset of patients with chronic pain who show an insufficient response to conventional analgesic treatments, it may be reasonable to consider the potential contribution of neurodevelopmental traits.

Current pharmacological management of chronic pain commonly includes tricyclic antidepressants, SNRIs, and gabapentinoids (79). Nevertheless, a substantial proportion of patients do not achieve adequate symptom relief using these approaches (4). This therapeutic gap suggests that additional perspectives may be warranted, particularly in patients presenting with neurodevelopmental characteristics such as ADHD traits.

In patients with chronic pain and co-occurring ADHD traits, alternative therapeutic perspectives may be considered. Several case reports and observational studies identified in this review have described pain improvement following ADHD-targeted pharmacological treatment (16, 26, 62). However, the available evidence remains preliminary and is largely based on uncontrolled studies, with very limited randomized evidence. Therefore, the effectiveness of ADHD medications for chronic pain remains uncertain.

Simultaneously, the reported associations between ADHD traits and pain severity (43, 68), along with neuroimaging findings (23), raise the possibility that central regulatory mechanisms may play a relevant role in certain subtypes of chronic pain.

Several priorities for future research emerge from the current literature. First, prospective studies are needed to determine the prevalence of ADHD traits in patients with chronic pain who are refractory to conventional treatment. Second, the efficacy and safety of ADHD-targeted pharmacological interventions in individuals with comorbid chronic pain should be evaluated in controlled clinical trials. Third, neuroimaging and neurophysiological markers should be investigated as potential predictors of treatment response.

Advances in these areas may contribute to the development of more individualized treatment strategies for specific chronic pain subtypes.

### 4.4 Limitations

Several limitations should be considered while interpreting the findings of this scoping review.

First, most of the included studies had a cross-sectional design, which precludes the determination of causal relationships or temporal sequencing between ADHD and chronic pain. Although large epidemiological studies have demonstrated associations between the two conditions, it remains unclear whether ADHD contributes to the development or persistence of chronic pain, whether chronic pain-related stress or sleep disturbances affect attentional functioning, or whether shared underlying mechanisms account for both. Hence, clarification of the causal pathways requires prospective longitudinal studies and RCTs.

Second, substantial heterogeneity exists in the methods used to assess ADHD across the studies. Many investigations relied on self-report screening instruments, such as the ASRS or WURS, without confirmation through structured diagnostic interviews. Self-reported measures may be subject to recall bias, and symptoms related to depression, anxiety, or cognitive impairment associated with chronic pain may overlap with ADHD symptomatology, potentially leading to misclassification.

Third, the potential influence of confounding factors should be considered. Depression and anxiety frequently co-occur with both ADHD and chronic pain (80, 81) and may partially mediate the observed associations. In addition, the use of analgesics or psychotropic medications may influence cognitive functioning and symptom reporting, complicating interpretations of the findings. Previous studies, including recent studies conducted by our research group (20, 82), have reported varying patterns of how ADHD symptoms and psychological factors contribute to pain severity. Although some analyses indicate a stronger direct association between ADHD symptoms and pain outcomes, other studies report that anxiety and depression as well as pain catastrophizing may exert substantial effects, including indirect influences, on pain severity. This variation highlights the complex and multifactorial nature of the relationship between ADHD and chronic pain. These factors may not only confound this association but may also represent shared underlying mechanisms.

Fourth, sampling bias may be present. Many clinical studies were conducted in tertiary care settings, where more severe or treatment-resistant cases are likely to be overrepresented. Furthermore, sex distribution varied across the study populations. For example, fibromyalgia samples predominantly included female patients, whereas ADHD was more commonly diagnosed in male participants (83). In some studies, sex differences were not systematically examined, which limits the interpretation of potential sex-specific effects.

Fifth, a considerable proportion of the included studies originated from a limited number of research groups. This likely reflects the emerging nature of the field and the concentration of research within specific regions or specialized centers. Replication by independent research groups across diverse geographical settings is important to strengthen the evidence base.

Finally, consistent with the nature of scoping reviews, we did not conduct a formal risk-of-bias assessment of individual studies. Additionally, only works published in English were included, thereby excluding the findings of relevant studies published in other languages.

Despite these limitations, this review provides a structured synthesis of previously fragmented findings and clarifies key hypotheses and research priorities for future investigations.

## 5 Conclusion

This scoping review indicates that evidence supporting an association between ADHD and chronic pain has been reported across diverse geographical regions and age groups. ADHD traits appear to be associated with greater pain severity and functional impairment, and higher prevalence rates have been reported in patients with treatment-resistant chronic pain.

From a clinical perspective, attention to neurodevelopmental features, such as fluctuating attention and restlessness, may contribute to a more comprehensive evaluation of individuals with chronic pain. Case reports describing improvements in pain outcomes following treatments targeting the dopaminergic and noradrenergic systems raise the possibility of shared neurobiological mechanisms between these two conditions.

However, most of the current evidence is derived from observational studies and case reports, and definitive conclusions regarding treatment efficacy or causal relationships cannot be drawn. Future prospective longitudinal studies and RCTs are required to systematically investigate the pathophysiology of and optimal treatment strategies for chronic pain comorbid with ADHD. These findings should be interpreted in the context of heterogeneous study designs and evidence that is predominantly observational.

## Data Availability

The original contributions presented in the study are included in the article/**Supplementary Material**. Further inquiries can be directed to the corresponding author.

## Supplementary material

Supplementary File 1 Detailed search strategy. This file contains the complete search strings and keywords used for each database (PubMed, PsycINFO, and Cochrane Library) to ensure study reproducibility.

Supplementary File 2 Supplementary Figures. This file contains additional figures illustrating publication trends, geographic distribution of included studies, and the distribution of therapeutic interventions reported in the literature included in this scoping review.

Supplementary File 3 PRISMA-ScR checklist. This checklist confirms that the scoping review adheres to the Preferred Reporting Items for Systematic reviews and Meta-Analyses extension for Scoping Reviews (PRISMA-ScR) guidelines.
