# Supplementary material for: Attention-deficit/hyperactivity disorder and chronic pain: a scoping review of epidemiology, clinical phenotypes, mechanisms, and treatment
Source: Front Psychiatry. 2026 Jul 13;17:1837517. doi: 10.3389/fpsyt.2026.1837517 (PMC13403107; doi:10.3389/fpsyt.2026.1837517)
Supplement: Supplementary File 1 — Detailed search strategy. This file contains the complete search strings and keywords used for each database (PubMed, PsycINFO, and Cochrane Library) to ensure study reproducibility. [file Supplementaryfile1.docx]

**Supplementary File 1**

**Supplementary Methods: Search Strategy**

The literature search strategy was developed with reference to previously published scoping reviews examining the relationship between attention-deficit/hyperactivity disorder (ADHD) and pain and was expanded to capture a broad range of diagnostic terminology, clinical concepts, and commonly used assessment instruments.

Electronic searches were conducted in PubMed, PsycINFO, and the Cochrane Library from database inception to December 28, 2025.

The search strategy consisted of two primary concept blocks combined using the Boolean operator AND: (1) an ADHD-related block and (2) a pain-related block. Within each block, controlled vocabulary terms (e.g., MeSH terms) and free-text keywords were combined using OR.

The ADHD-related block included the MeSH term “attention deficit disorder with hyperactivity” together with a broad range of title and abstract keywords representing contemporary and historical diagnostic terminology and commonly used diagnostic interviews and rating scales.

The pain-related block included the MeSH term “pain” and related controlled vocabulary terms describing pain conditions and syndromes, together with free-text keywords capturing a wide range of pain-related symptoms and diagnoses.

Search strategies were adapted as appropriate for each database. The complete electronic search strategy used for PubMed is presented below.

**PubMed Search Strategy**

**ADHD-related search block**

#1

("Attention Deficit Disorder with Hyperactivity"[MeSH] OR ADHD*[tiab] OR ADDH[tiab] OR ADHS[tiab] OR ADSH[tiab] OR "AD/HD"[tiab] OR HKD[tiab] OR MBD[tiab] OR (Attention*[tiab] AND (Deficit*[tiab] OR impair*[tiab] OR problem*[tiab])) OR attention-deficit*[tiab] OR (("Minimal Cerebral"[tiab] OR "Minimal Brain"[tiab]) AND (damage*[tiab] OR dyfunc*[tiab] OR disorder*[tiab])) OR ((hyperkine*[tiab] OR overactive*[tiab] OR hyperactiv*[tiab]) AND (syndrome*[tiab] OR disorder*[tiab])) OR ASRS-Adolescent[tiab] OR SWAN[tiab] OR AIM-C[tiab] OR DAYAS*[tiab] OR SKAMP[tiab] OR SNAP-IV[tiab] OR SNAP-V[tiab] OR VADRS[tiab] OR "Conners Rating Scale"[tiab] OR "Conners 3"[tiab] OR "Conners-Wells"[tiab] OR "Weiss Functional Impairment Rating Scale"[tiab] OR WFIRS[tiab] OR (("Child Behavior Checklist"[tiab] OR CBCL[tiab]) AND "Attention Problem"[tiab]) OR (("K-SADS"[tiab] OR KSADS[tiab] OR "Kiddie Schedule for Affective Disorders and Schizophrenia"[tiab]) AND attention[tiab]) OR ("C-DISC"[tiab] AND attention[tiab]) OR "Adult ADHD Self-Report Scale"[tiab] OR ASRS[tiab] OR "ASRS v1.1"[tiab] OR CAARS[tiab] OR "Conners' Adult ADHD Rating Scale"[tiab] OR AISRS[tiab] OR "Adult ADHD Investigator Symptom Rating Scale"[tiab] OR WURS[tiab] OR "Wender Utah Rating Scale"[tiab] OR BAARS[tiab] OR "Barkley Adult ADHD Rating Scale"[tiab] OR DIVA[tiab] OR "Diagnostic Interview for ADHD in Adults"[tiab] OR CAADID[tiab] OR "Conners' Adult ADHD Diagnostic Interview for DSM-IV"[tiab])

**Pain-related search block**

#2

(Pain[MeSH] OR "Pain Clinics"[MeSH] OR "Pain Management"[MeSH] OR "Complex Regional Pain Syndromes"[MeSH] OR "Fibromyalgia"[MeSH] OR "Patellofemoral Pain Syndrome"[MeSH] OR "Temporomandibular Joint Dysfunction Syndrome"[MeSH] OR pain[tiab] OR pains[tiab] OR pained[tiab] OR paining[tiab] OR painful*[tiab] OR "Central Sensitivity Syndrome"[tiab] OR colic*[tiab] OR erythromelalg*[tiab] OR "Failed Back Surgery Syndrome"[tiab] OR fibromyalg*[tiab] OR "Interstitial Cystit*"[tiab] OR "Morton Neuroma*"[tiab] OR "Phantom Limb*"[tiab] OR "Piriformis Muscle Syndrome*"[tiab] OR "Reflex Sympathetic Dystroph*"[tiab] OR sciatica[tiab] OR "Slit Ventricle Syndrome*"[tiab] OR "Symphysis Pubis Dysfunction"[tiab] OR (temporomandibular[tiab] AND dysfunc*[tiab]) OR vaginismus[tiab] OR vulvodyn*[tiab] OR abdominalg*[tiab] OR acrostealg*[tiab] OR alge*[tiab] OR algi*[tiab] OR algol*[tiab] OR algom*[tiab] OR angina[tiab] OR anginal[tiab] OR arthralg*[tiab] OR arthritis*[tiab] OR causalgia*[tiab] OR cephalg*[tiab] OR dyspeps*[tiab] OR dysmenorrhe*[tiab] OR dysur*[tiab] OR earache*[tiab] OR enterodyn*[tiab] OR esophagodyn*[tiab] OR gastralg*[tiab] OR glossalg*[tiab] OR hyperalges*[tiab] OR mastodyn*[tiab] OR metatarsalg*[tiab] OR mastalg*[tiab] OR myalg*[tiab] OR neuralg*[tiab] OR neuropath*[tiab] OR nocicept*[tiab] OR odontalg*[tiab] OR omalg*[tiab] OR ostalg*[tiab] OR otalg*[tiab] OR prosopalg*[tiab] OR rachialg*[tiab] OR toothache*[tiab] OR urethrodyn*[tiab])

**Final search combination**

#1 AND #2
