# Supplementary material for: Attention-deficit/hyperactivity disorder and chronic pain: a scoping review of epidemiology, clinical phenotypes, mechanisms, and treatment
Source: Front Psychiatry. 2026 Jul 13;17:1837517. doi: 10.3389/fpsyt.2026.1837517 (PMC13403107; doi:10.3389/fpsyt.2026.1837517)
Supplement: Supplementary File 2 — Supplementary Figures. This file contains additional figures illustrating publication trends, geographic distribution of included studies, and the distribution of therapeutic interventions reported in the literature included in this scoping review. [file Supplementaryfile2.docx]

**Supplementary File 2**

**Supplementary Figures**

**Supplementary Figure 1. Annual Publication Trends (2006–2025)
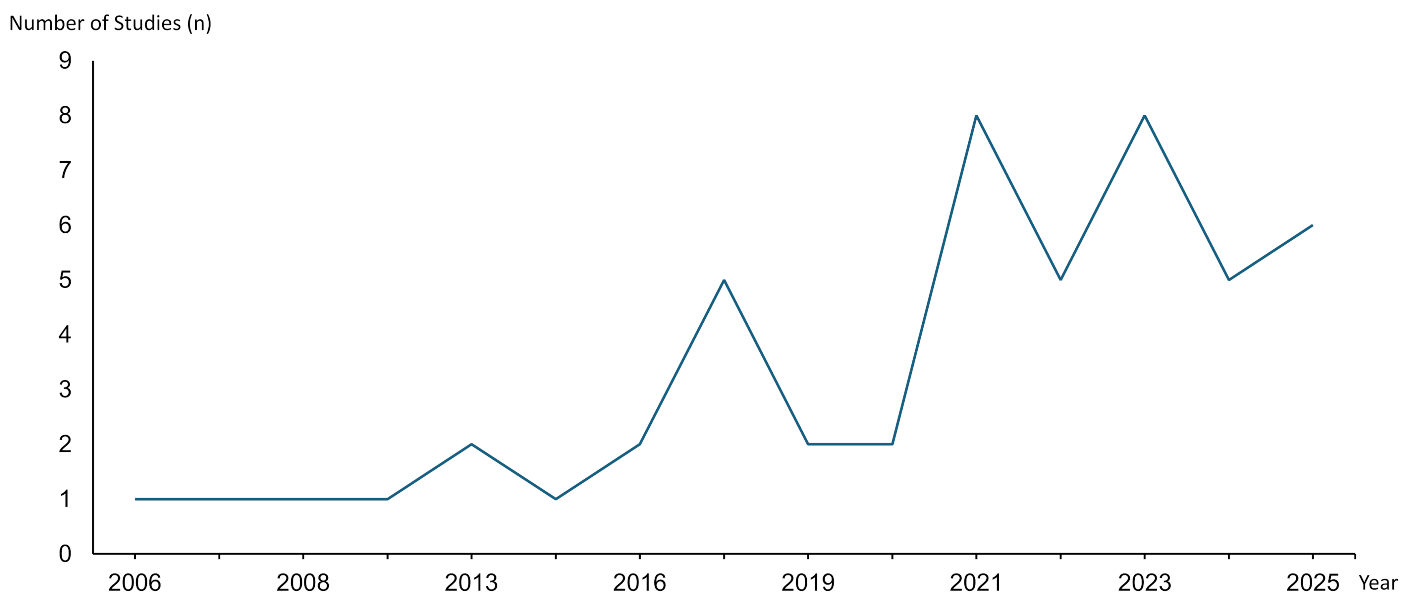
**

The line graph illustrates the annual number of publications included in this review. The earliest reports appeared in 2006–2007, with a noticeable increase in publications after 2018. This trend suggests growing research interest in the comorbidity of ADHD and chronic pain in recent years.

**Abbreviations:** ADHD, attention-deficit/hyperactivity disorder.


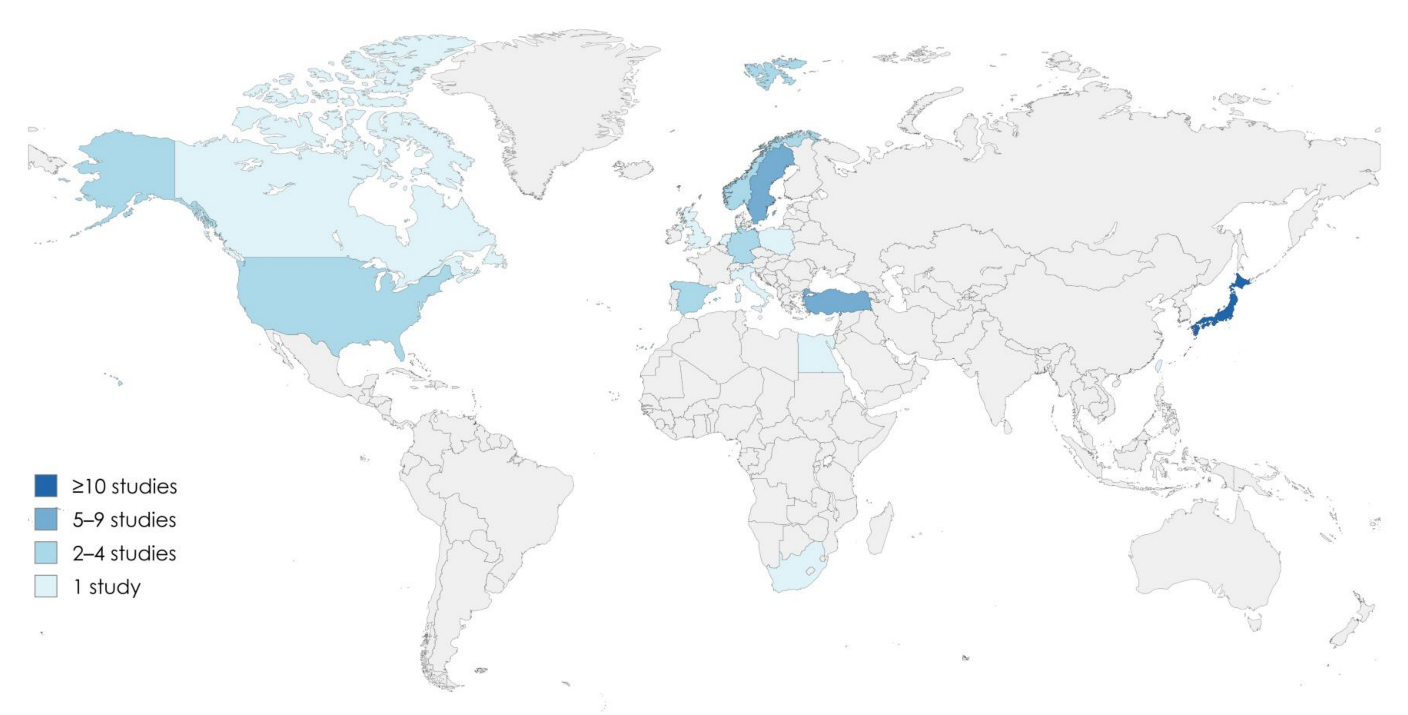
**Supplementary Figure 2. Geographic Distribution of Included Studies**

This world map illustrates the number of included studies by country. The largest number of studies were conducted in Japan (n = 15, 30.0%), followed by Sweden (n = 9, 18.0%) and Turkey (n = 6, 12.0%). Additional studies were conducted in Norway (n = 4, 8.0%) and the United States (n = 4, 8.0%), while two studies each were conducted in Spain (n = 2, 4.0%) and Germany (n = 2, 4.0%). One study each (2.0%) was conducted in the Netherlands, Canada, the United Kingdom, South Africa, Taiwan, Italy, Poland, and Egypt.


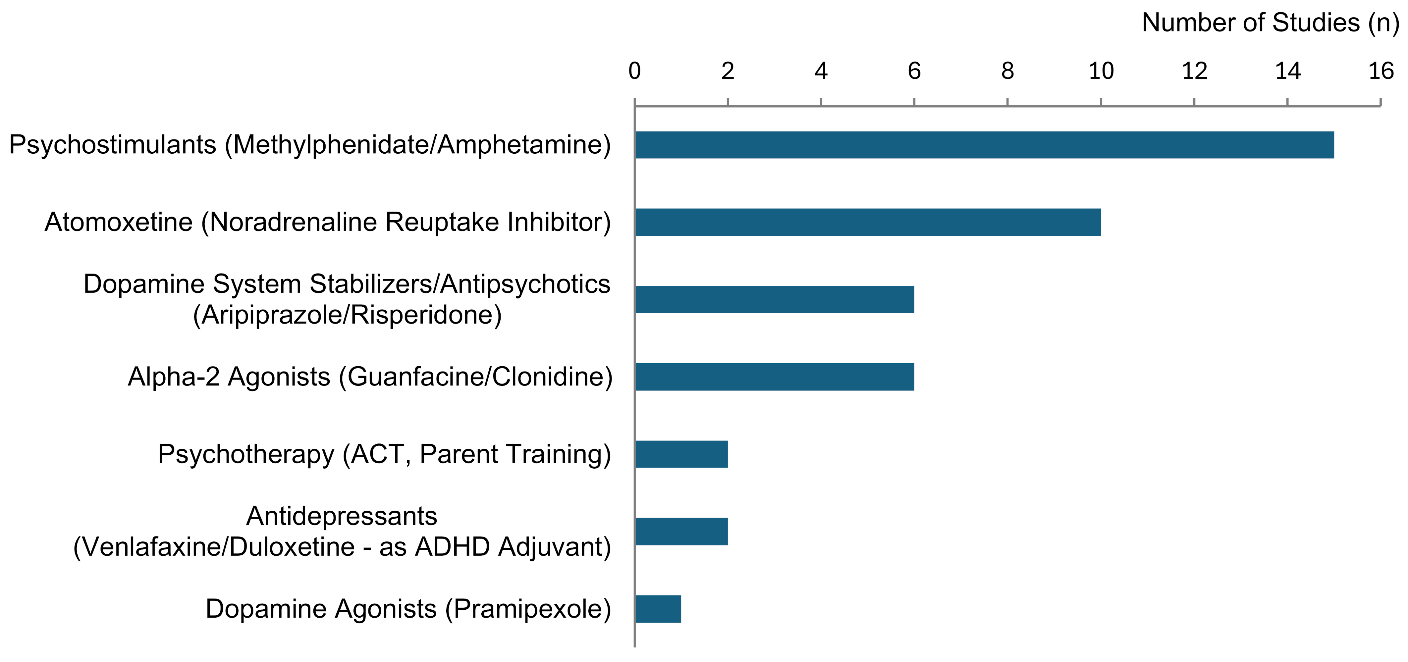
**Supplementary Figure 3. Distribution of Therapeutic Interventions in the Included Studies**

This bar graph summarizes the primary intervention types or pharmacological classes used in studies that reported therapeutic interventions (n = 18). As some studies reported multiple interventions, the counts are not mutually exclusive. Psychostimulants (methylphenidate or amphetamines) were reported most frequently (n = 15), followed by atomoxetine (n = 10), dopamine system stabilizers or antipsychotics (e.g., aripiprazole; n = 6), and α2-adrenergic agonists (e.g., guanfacine; n = 6). Psychological interventions were less commonly reported, with one study describing acceptance and commitment therapy (ACT) for patients and another reporting ACT-based parent training.

**Abbreviations:** ADHD, attention-deficit/hyperactivity disorder.
